# An interplay between multiple sirtuins promotes completion of DNA replication in cells with short telomeres

**DOI:** 10.1371/journal.pgen.1007356

**Published:** 2018-04-16

**Authors:** Antoine Simoneau, Étienne Ricard, Hugo Wurtele

**Affiliations:** 1 Centre de recherche de l’Hôpital Maisonneuve-Rosemont, boulevard de l’Assomption, Montréal, Canada; 2 Programme de Biologie Moléculaire, Université de Montréal, Montréal, Canada; 3 Département de Médecine, Université de Montréal, Montréal, Canada; Chinese Academy of Sciences, CHINA

## Abstract

The evolutionarily-conserved sirtuin family of histone deacetylases regulates a multitude of DNA-associated processes. A recent genome-wide screen conducted in the yeast *Saccharomyces cerevisiae* identified Yku70/80, which regulate nonhomologous end-joining (NHEJ) and telomere structure, as being essential for cell proliferation in the presence of the pan-sirtuin inhibitor nicotinamide (NAM). Here, we show that sirtuin-dependent deacetylation of both histone H3 lysine 56 and H4 lysine 16 promotes growth of *yku70Δ* and *yku80Δ* cells, and that the NAM sensitivity of these mutants is not caused by defects in DNA double-strand break repair by NHEJ, but rather by their inability to maintain normal telomere length. Indeed, our results indicate that in the absence of sirtuin activity, cells with abnormally short telomeres, e.g., *yku70/80Δ* or *est1/2Δ* mutants, present striking defects in S phase progression. Our data further suggest that early firing of replication origins at short telomeres compromises the cellular response to NAM- and genotoxin-induced replicative stress. Finally, we show that reducing H4K16ac in *yku70Δ* cells limits activation of the DNA damage checkpoint kinase Rad53 in response to replicative stress, which promotes usage of translesion synthesis and S phase progression. Our results reveal a novel interplay between sirtuin-mediated regulation of chromatin structure and telomere-regulating factors in promoting timely completion of S phase upon replicative stress.

## Author summary

Proliferating cells duplicate their genetic material via a highly-ordered process called DNA replication. Genetic lesions caused by a variety of environmental chemicals can inhibit DNA replication progression, thereby causing genetic abnormalities and cell death, as well as promoting the development of diseases such as cancer. To fit within the confines of the cell’s nucleus, DNA is wrapped around proteins called histones, which play critical roles in promoting accurate DNA replication. In this study, we reveal unexpected functional links between histones and cellular factors that regulate telomeres, which are structures protecting the extremities of DNA molecules in cells. We demonstrate that a functional interplay between histone- and telomere-regulating factors allow cells to duplicate their genetic material in a timely manner in the presence of replication-blocking chemicals. Overall, our study highlights new mechanisms through which proliferating cells avoid the deleterious consequences associated with compromised DNA replication.

## Introduction

Histone post-translational modifications influence chromatin structure and serve as recruitment platforms for diverse protein complexes [1]. Acetylation of histones on lysine residues is catalysed by histone acetyltransferases (HAT) and reversed by histone deacetylases (HDAC). Four HDAC classes are defined based on sequence identity and catalytic mechanism [2]. Class III HDACs are referred to as sirtuins because of their sequence homology to yeast Sir2. These enzymes deacetylate lysine residues in histone and non-histone proteins in a reaction that requires nicotinamide adenine dinucleotide (NAD^+^) and releases nicotinamide and O-acetyl ADP ribose [3,4]. Sirtuins are evolutionarily conserved, and regulate several DNA-associated processes including gene silencing, DNA replication, and DNA repair [5].

The genome of the budding yeast *Saccharomyces cerevisiae* encodes 5 sirtuins: Sir2 and Homolog of Sir Two (Hst) 1–4 [6,7]. Sir2-dependent deacetylation of histone H4 lysine 16 (H4K16ac) controls gene silencing at the yeast mating and ribosomal DNA (rDNA) loci [7,8] as well as at telomeres [9], and modulates replicative lifespan [10,11]. Hst1 regulates sporulation gene expression [12,13], and also controls thiamine biosynthesis and intracellular NAD^+^ levels at the transcriptional level [14,15]. Hst2 displays partial functional redundancy with Sir2 as its overexpression can rescue silencing defects in *sir2Δ* mutants [16,17]. Hst3 and Hst4 reverse histone H3 lysine 56 acetylation (H3K56ac) [18], a modification catalyzed by the HAT Rtt109 on virtually all newly synthesized histones in yeast [19,20]. H3K56ac-harboring nucleosomes are assembled behind DNA replication forks to maintain appropriate nucleosomal density on daughter chromatids following parental histone segregation, and are deacetylated genome-wide by Hst3/4 during the G2/M phase. Cells lacking both Hst3 and Hst4 present constitutive H3K56ac throughout the cell cycle, which causes severe phenotypes including spontaneous DNA damage, chromosomal instability, elevated replicative stress and DNA damage-induced signaling, as well as extreme sensitivity to high temperature and drugs that impede DNA replication [18,21,22]. However, the precise molecular mechanisms by which Hst3/4-mediated H3K56ac deacetylation promotes resistance to replicative stress remain unclear.

The kinases Mec1 and Rad53 are activated during replicative stress to phosphorylate multiple substrates which cooperate to inhibit DNA replication origin activation, stabilize stalled replication forks, and increase dNTP pools [23]. Rad53 activation depends on the mediator protein Rad9, which is recruited to chromatin through interaction with phosphorylated serine 128 of histone H2A (γ-H2AX, a DNA damage-induced modification) and methylated histone H3 lysine 79 (H3K79me) [24–27], the latter being catalyzed by the methyltransferase Dot1 [28,29]. Recent data demonstrate that cells have evolved mechanisms that limit Rad53 activation upon replicative stress, as well as others that permit its progressive inactivation upon DNA lesion resolution [30–32]. While the precise consequences of Rad53 “hyperactivation” are incompletely characterized, its biological relevance is highlighted by the fact that it causes sensitivity to replicative stress-inducing drugs [31]. Interestingly, we and others have shown that limiting Rad53 activation via *DOT1* deletion or histone gene mutations that inhibit H3K79me promotes resistance to DNA replication-blocking drugs in several yeast mutants, including *hst3Δ hst4Δ* cells, by elevating usage of error-prone translesion synthesis (TLS) [22,33–35]. These observations emphasize the importance of the interplay between chromatin and DNA damage checkpoint signalling in regulating the cellular response to replicative stress.

In eukaryotes, DNA replication is initiated in a temporally ordered manner at genomic regions called “origins” that are activated in early, mid, or late S phase [36]. Genomic context influences the timing of origin activation (or “firing”); for example, in the yeast *S*. *cerevisiae* origins located near telomeres and within rDNA repeats are activated during late S, while those close to centromeres fire earlier [37–40]. Interestingly, the silent information regulator (SIR) HDAC complex, which comprises the Sir2-Sir3-Sir4 subunits and deacetylates telomeric/subtelomeric chromatin, has been shown to prevent early firing of telomeric origins [41]. Telomere length also influences origin activity; indeed, cells with short telomeres, such as those lacking the Yku70/80 complex, initiate DNA replication at telomeric and subtelomeric regions abnormally early during S phase [42–44]. While several telomere-associated factors have been shown to influence the timing of telomeric DNA replication origin [43–46], the functional significance of such regulation is poorly understood.

The Yku70/80 complex is present at chromosomal ends where it protects telomeres from nucleolytic degradation and promotes recruitment of telomerase. Cells lacking Yku70/80 heterodimers present short but stable telomeres harboring abnormally long stretches of ssDNA [47–51]. Yku70/80 is also involved in DNA double-strand break (DSB) repair by non-homologous end joining (NHEJ). This complex binds DSB ends where it recruits the DNA ligase machinery composed of Lif1-Dnl4 and Nej1, thereby promoting end ligation [47,52,53]. Interestingly, cells lacking Yku70/80 are sensitive to genotoxins that generate DNA replication-blocking lesions without directly causing DSBs, suggesting an NHEJ-independent role for this complex during replicative stress [54,55]. However, the extent to which the other cellular functions of Yku70/80, e.g., at telomeres, might influence the cellular response to DNA replication stress is unclear.

A genetic screen conducted by our group in *S*. *cerevisiae* identified *yku70Δ* and *yku80Δ* mutants as sensitive to pharmacological inhibition of sirtuin HDACs by nicotinamide (NAM) [35]. Since NAM causes replicative stress [35], we originally postulated that the Yku70/80 complex might influence DNA replication progression in the absence of sirtuin activity. Here, we reveal that a novel interplay between multiple sirtuins promotes completion of DNA replication in *yku70Δ* and *yku80Δ* cells, and that telomere shortening is the root cause of the sensitivity of these mutants to NAM-induced sirtuin inhibition. Our data further indicate that misregulation of replication origin firing at short telomeres, as well as modulation of DNA damage checkpoint kinase activity by chromatin structure, influence the resistance to NAM- and genotoxin-induced replicative stress in cells with short telomeres.

## Results

### An interplay between multiple sirtuins permits growth of cells lacking Yku70/80

**Fig 1:**
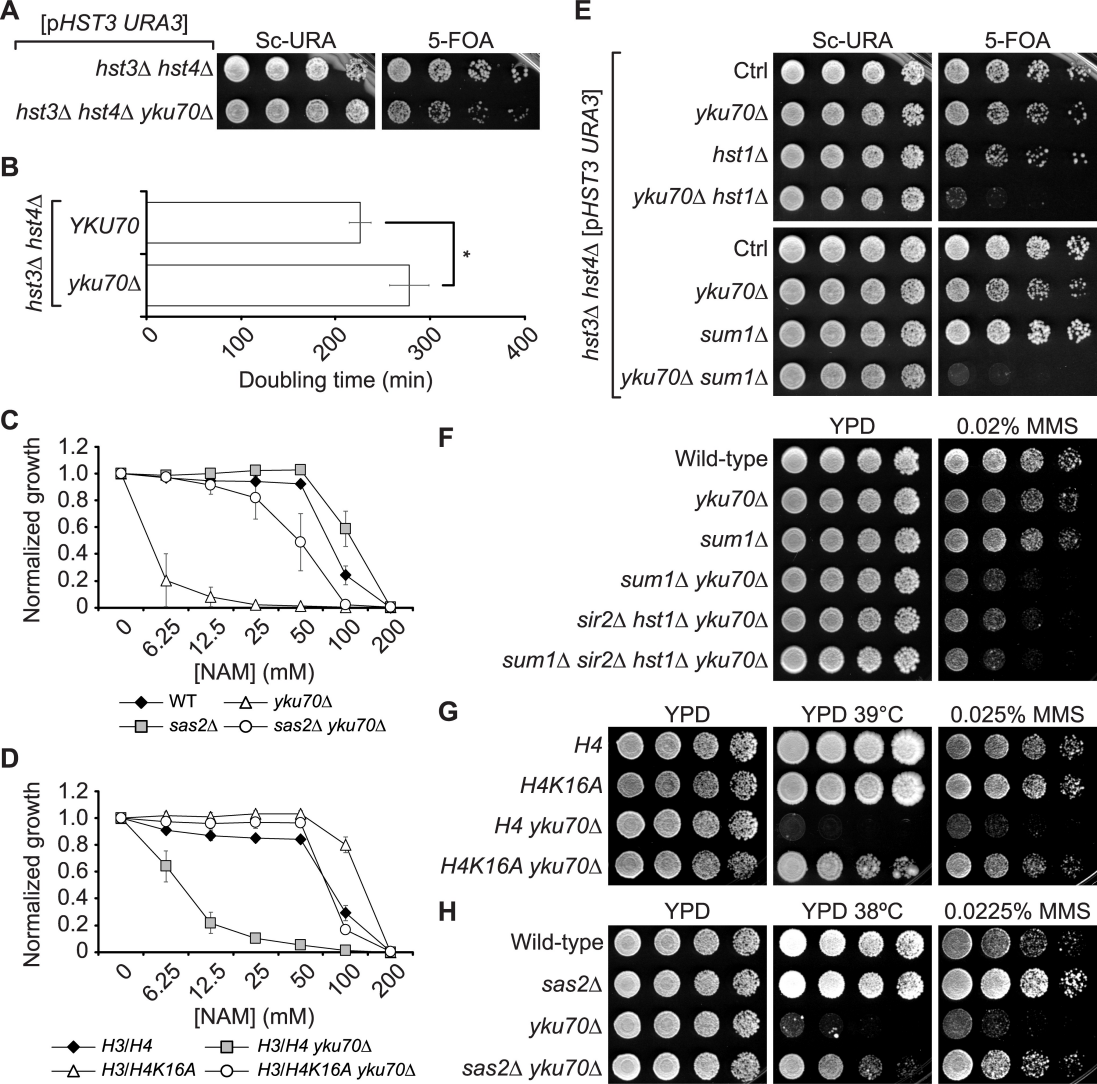
Multiple sirtuins permit growth of cells lacking Yku70/80. (A-B) *yku70Δ* causes synthetic growth defects when combined with *hst3Δ hst4Δ*. Five-fold serial dilution of cells were spotted on solid media and incubated at 25°C. (B) Doubling time for strains in A incubated in YPD at 30°C (see material and methods). Error bars: standard deviation, p-value = 7.42x10^-7^ (two-sided student’s T-test). (C-D) Preventing H4K16 acetylation rescues the growth of *yku70Δ* mutants in NAM. Yeast cells were incubated in a 96-well plate containing increasing concentrations of NAM. OD_630_ readings were acquired after 48 h at 30°C and results were normalized to untreated controls. Error bars: standard deviation. (E) Deletion of genes encoding subunits of the Hst1-Sum1-Rfm1 complex causes synthetic lethality when combined with *hst3Δ hst4Δ yku70Δ*. (F) Lack of Sum1 causes synthetic sensitivity to MMS-induced replicative stress when combined *yku70Δ*. (G-H) H4K16ac is deleterious to the growth of *yku70Δ* mutants in the presence of MMS-induced replicative stress or at elevated temperatures.

*S*. *cerevisiae yku70Δ* and *yku80Δ* mutants are sensitive to NAM [35], a pan-sirtuin inhibitor [3,56]. To identify which among the five yeast sirtuins (Sir2, Hst1-4) are responsible for this phenomenon, single deletions of each sirtuin gene were combined with *yku70Δ* by mating, and double mutants isolated via tetrad dissection (S1A Fig). None of the double mutants displayed noticeable growth defects, suggesting that NAM-induced growth inhibition in *yku70Δ* mutants is likely due to concurrent inhibition of multiple sirtuins. We previously showed that deletion of the H3K56ac acetyltransferase *RTT109* rescues the sensitivity of *yku70Δ* and *yku80Δ* mutants to NAM [35]. We therefore tested whether this reflects NAM-induced inhibition of the H3K56ac-deacetylases Hst3 and Hst4 [18]. Consistently, we found that *yku70Δ hst3Δ hst4Δ* cells displayed moderate but significant decrease in growth rate and doubling time compared to *hst3Δ hst4Δ* cells (Fig 1A and 1B), and that the *H3K56A* mutation, which prevents H3K56ac, allows proliferation of *yku70Δ* cells in NAM (S1B and S1C Fig). Nevertheless, growth of the *yku70Δ hst3Δ hst4Δ* mutant could still be significantly exacerbated by exposure to NAM (S2A Fig). Thus, while H3K56 hyperacetylation is an essential component of the NAM sensitivity of *yku70Δ* mutants, inhibition of sirtuins other than Hst3/4 also probably contribute to this phenomenon.

Mutations inhibiting H4K16 acetylation (H4K16ac), the levels of which are regulated by Sir2 and Hst1 *in vivo* [57,58], partially rescue certain phenotypes of *hst3Δ hst4Δ* cells [22]. Interestingly, deletion of *SAS2*, a gene encoding the catalytic subunit of the H4K16 acetyltransferase complex SAS-I [57,59,60], or mutation of H4K16 to alanine (H4K16A), rescued growth of *yku70Δ* cells in NAM (Fig 1C and 1D). We could not directly test whether reduced Sir2 activity exacerbates the growth defects of *yku70Δ hst3Δ hst4Δ* mutants since *sir2Δ* causes synthetic lethality when combined with *hst3Δ hst4Δ* [22,61]. Sir2 is recruited to rDNA repeats and *HMR*/*HML*/telomeres as part of either the RENT (Sir2/Cdc14/Net1) or SIR (Sir2/Sir3/Sir4) complexes, respectively [62–64]. We could not evaluate the impact of RENT subunit-encoding *CDC14* or *NET1* genes on *hst3Δ hst4Δ* cells since their deletion causes lethality. rDNA silencing defects arising from lack of Sir2 activity in the context of the RENT complex can be rescued by deletion of *FOB1*, which encodes a component of the rDNA replication fork barrier [10,65,66]. We found that deletion of *FOB1* did not rescue the sensitivity of *yku70Δ* cells to NAM (S1D Fig); moreover, combining *hst3Δ hst4Δ yku70Δ* with either *sir3Δ* or *sir4Δ* did not cause synthetic growth defects (S1E Fig). Overall these results suggest that growth of *yku70Δ* cells depends on Hst3/4 and either i) on sirtuins other than Sir2, or ii) on Sir2-dependent processes that are not associated with the RENT or SIR complexes.

The Hst1-Sum1-Rfm1 complex promotes H4K16ac deacetylation *in vivo* [58]. Interestingly, deletion of either *HST1* or *SUM1* provoked synthetic growth defects when combined with *hst3Δ hst4Δ yku70Δ* (Fig 1E). Since constitutive hyperacetylation of H3K56 in *hst3Δ hst4Δ* mutants causes spontaneous DNA damage [18], we reasoned that elevated sensitivity to replicative stress in cells lacking both Hst1-Sum1-Rfm1 and Yku70/80 might explain the observed synthetic lethality. Consistently, deletion of *SUM1* sensitized *yku70Δ* mutants to the DNA alkylating agent methylmethane sulfonate (MMS; Fig 1F). This was not the case for *hst1Δ*, implying that Hst1-independent Sum1 functions might influence growth of *yku70Δ* cells in MMS (S2B Fig). Published data indicate that Sir2 interacts with Sum1 to promote transcriptional silencing in the absence of Hst1 [67]. While deletion of *SIR2* did not confer increased MMS sensitivity in *yku70Δ* mutants, the *sir2Δ hst1Δ yku70*Δ triple mutant was strongly sensitized to MMS compared to control double mutants (Fig 1F, S2B Fig). Moreover, the MMS sensitivity of *sir2Δ hst1Δ yku70Δ* was similar to that of *sum1Δ yku70Δ*, and was not further increased in *sir2Δ hst1Δ sum1Δ yku70*Δ cells (Fig 1F). We also note that *sir2Δ hst1Δ yku70Δ* mutants remain sensitive to NAM (S2C Fig), consistent with the notion that concurrent inhibition of multiple sirtuins, i.e., Sir2, Hst1, Hst3 and Hst4, causes the sensitivity of *yku70Δ* cells to this agent.

We next tested whether preventing H4K16 acetylation alleviates other phenotypes of cells lacking Yku70/80. In addition to their MMS sensitivity, *yku70Δ* mutants exhibit growth and DNA replication defects at elevated temperatures [68]. We found that the *H4K16A* and *sas2Δ* mutations rescued the MMS sensitivity of *yku70Δ* and *yku70Δ sum1Δ* mutants (Fig 1G and 1H, S2D Fig). The temperature sensitivity of *yku70Δ* cells could also be suppressed by mutations that reduce H4K16ac levels (Fig 1G and 1H, S2D Fig), and was exacerbated by *sum1Δ* in a H4K16ac-dependent manner (S2D Fig). Overall, our data indicate that the inability of *yku70Δ* mutants to grow in the presence of NAM results in part from lack of Sum1-Hst1/Sir2 activity, which leads to misregulation of H4K16ac levels and consequent sensitization to replicative stress caused by constitutive H3K56ac.

### Short telomeres sensitize cells to NAM-induced sirtuin inhibition

**Fig 2:**
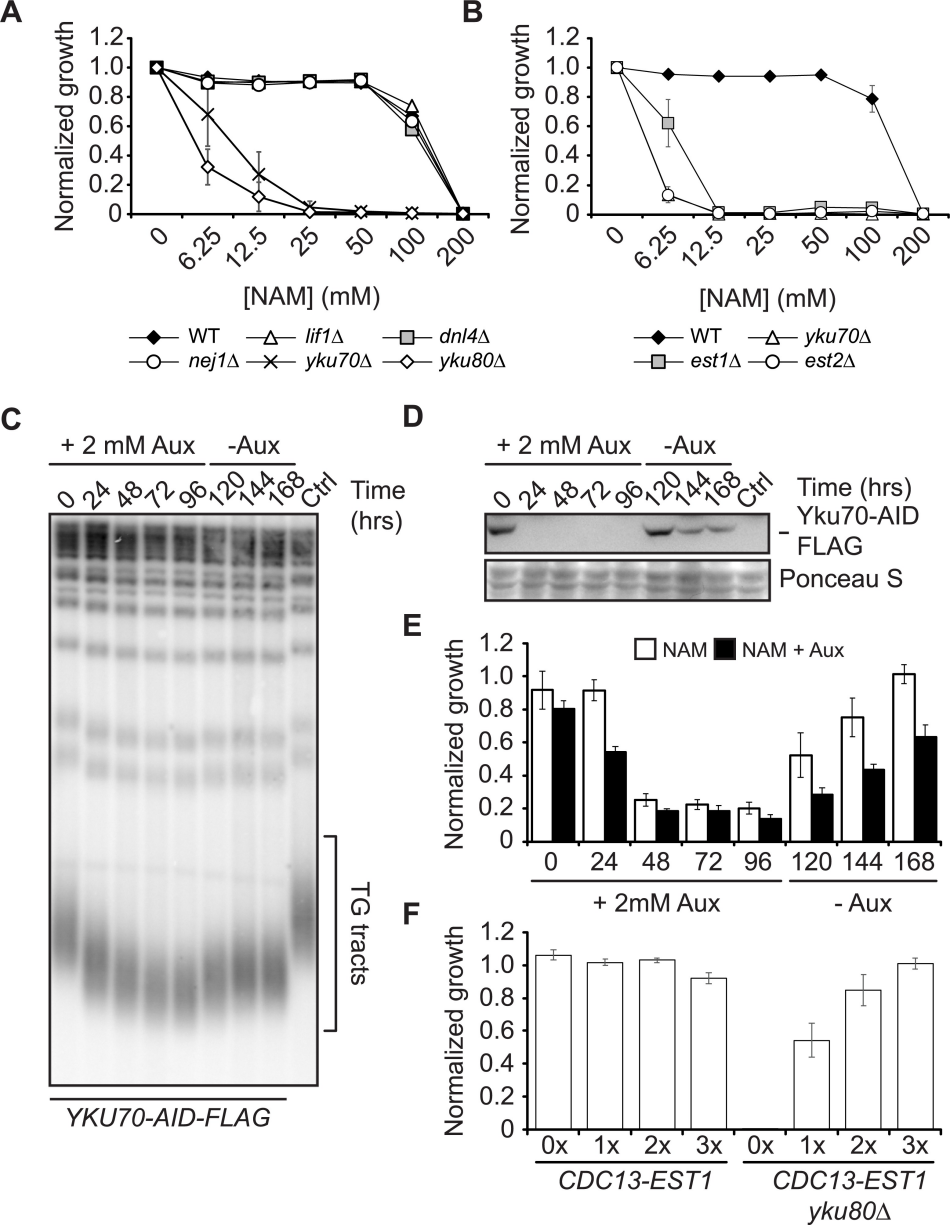
Growth defects of *yku70Δ* mutants in NAM result from telomere shortening. (A) Lack of NHEJ does not cause growth defects in NAM. (B) Telomerase mutants display severe growth defects in NAM. (A-B) Growth assay in 96-well plates (see materials and methods). Error bars: standard deviation. (C-E) Reduction of telomere length associated with Yku70 depletion causes NAM-induced growth defects. Yku70-AID-Flag-expressing yeasts were incubated in YPD at 30°C in the presence of auxin for 4 days to degrade Yku70. Cells were then transferred to YPD media without auxin to allow Yku70 re-expression. (C) Southern blot analysis of telomere length. (D) Yku70 degradation and re-expression was monitored by immunoblotting. (E) Samples were taken at every time point to evaluate cell growth in 12.5 mM NAM with or without auxin for 24h. Error bars: Standard deviation. (F) Increasing telomere length of the *yku80Δ* mutant rescues its growth in NAM. A plasmid expressing the Cdc13-Est1 fusion was transformed in *cdc13Δ* cells harboring a plasmid encoding *CDC13* and a *URA3* marker. Samples were taken at indicated re-streaking 1x, 2x or 3x) after 5-FOA counterselection to test growth in 12.5 mM NAM. OD readings were taken after 48 hours of growth at 30°C and normalized on untreated control. See S5 Fig for corresponding assessment of telomere length by southern blotting. Error bars: Standard deviation.

The Yku70/80 complex is required for both DNA repair by non-homologous end joining (NHEJ) and telomere maintenance [47,48]. NHEJ-abolishing mutations (*lif1Δ*, *nej1Δ* and *dnl4Δ*) did not cause notable growth defects in NAM (Fig 2A), indicating that the sensitivity of *yku70Δ*/*80Δ* mutants to this chemical is unlikely to result from defective DSB repair by NHEJ. Cells lacking Yku70/80 present very short, but stable, telomeres [48,49]. Pre-senescent haploid cells lacking the telomerase subunit-encoding genes *EST1* and *EST2* also present very short telomeres [47,69], and are as sensitive to NAM as *yku70Δ* cells (Fig 2B), suggesting that reduced telomere length might cause NAM sensitivity. On the other hand, the Yku70/80 complex promotes telomerase intracellular trafficking and recruitment to telomeres by binding to the TLC1 telomerase RNA [51,70–73], raising the possibility that defective recruitment of telomerase to telomeres, and not telomere length *per se*, might influence NAM sensitivity. Contrary to this notion, the *yku80-135i* mutation, which eliminates the TLC1-binding functions of Yku80, while only slightly reducing telomere length, did not sensitize cells to NAM (S3A and S3B Fig).

To further investigate the impact of telomere length on NAM sensitivity, we performed a time course experiment using a strain expressing an auxin-inducible degron (AID)-tagged *YKU70* allele [74,75]. Auxin addition to the growth medium provoked rapid (within one hour) degradation of Yku70 and progressive telomere shortening over several days of cell growth, while auxin removal allowed rapid Yku70 re-expression and progressive telomere length recovery (Fig 2C and 2D, S4 Fig). We reasoned that if Yku70 activity/presence within the cell is important for NAM resistance, sensitivity to this agent should increase within hours of auxin treatment. In contrast, if telomere length homeostasis promotes NAM resistance, progressive decrease in telomere length caused by Yku70 depletion should lead to a concomitant increase in NAM sensitivity over several days of growth. At every time point analyzed, we tested the capacity of cells to grow in NAM with or without auxin, *i*.*e*., with or without Yku70 re-expression during NAM exposure (Fig 2E). Strikingly, NAM sensitivity correlated well with overall telomere length of the cell population, and re-expression of Yku70 during the growth assay (by omitting auxin in NAM-containing medium) did not reverse this trend. Further supporting the notion that telomere length, and not presence of Yku70/80 *per se*, is important to promote survival in NAM, expression of a Cdc13-Est1 chimera that bypasses Yku70/80 in telomerase-mediated telomere elongation [51,76] rescued growth of *yku80Δ* mutants in NAM (Fig 2F, S5 Fig). Finally, mutation of *ELG1*, which was shown to extend telomeres et re-establish telomeric origin repression in *yku70Δ* cells [44], also rescued the growth of *yku70Δ* mutants in NAM (S6A Fig). We note that NAM did not significantly affect telomere length in conditions used for our experiments (S6B Fig). Together, our results indicate that telomere length is an important determinant of NAM sensitivity in cells lacking Yku70/80.

### Cells with short telomeres present Tel1-dependent DNA replication defects upon NAM exposure

**Fig 3:**
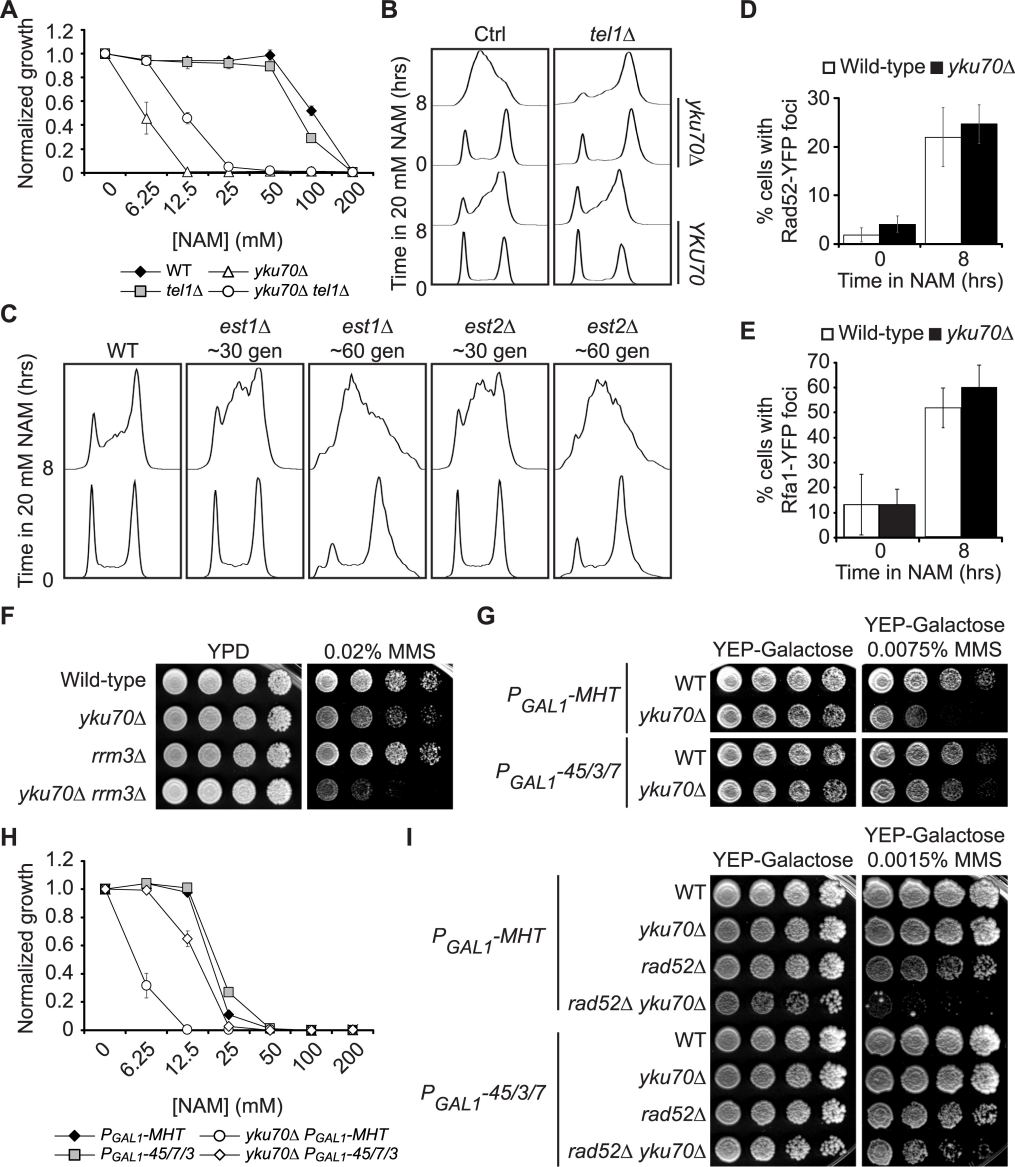
Cells with short telomeres present Tel1-dependent defects in completing DNA replication upon NAM exposure. (A) *tel1Δ* does not cause NAM sensitivity and rescues the growth of *yku70Δ* cells in NAM (B) *tel1Δ* rescues the S phase progression defects of *yku70Δ* mutants in NAM. (C) Cells lacking telomerase subunits arrest in S-phase upon NAM-exposure. (D-E) *yku70Δ* cells do not present increased proportion of cells with Rad52-YFP (D) or Rfa1-YFP (E) foci compared to wild-type upon NAM exposure. (B-E) Asynchronous cells were incubated in YPD for 8 hours at 30°C in the presence of 20 mM NAM. Samples were taken at indicated time for flow cytometry DNA content analysis or fluorescence microscopy (Rad52-YFP or Rfa1-YFP foci). (F) *rrm3Δ* exacerbates the MMS-induced replicative stress sensitivity of *yku70Δ* cells. (G-H) Galactose-induced overexpression of *CDC45*, *SLD3* and *SLD7* (45/3/7) improves the growth of *yku70Δ* mutants in MMS (G) and NAM (H). A construct expressing a Myc-His Tag (MHT) was used for the control condition. (I) Overexpression of *CDC45*, *SLD3* and *SLD7* (45/3/7) rescues the synthetic growth defects of *yku70Δ rad52Δ* mutants exposed to MMS.

Intriguingly, deletion of *TEL1*, despite rendering telomeres as short as those of *yku70Δ* or *est1/2Δ* mutants [77,78], did not provoke growth defects in NAM, and even rescued the NAM sensitivity of *yku70Δ* cells (Fig 3A). Telomeres of *yku70Δ* mutants, but not *tel1Δ*, are resected by the nuclease Exo1 and therefore accumulate ssDNA [49,79]. However, deleting *EXO1* did not eliminate the growth defects of *yku70Δ* mutants in NAM (S3C Fig), indicating that elevated ssDNA at telomeres cannot explain the differential sensitivity of *yku70Δ* vs *tel1Δ* cells to NAM. In contrast to *yku70Δ* and *yku80Δ* mutants, cells lacking Tel1 activate their telomeric replication origins in late S; moreover, deletion of *TEL1* restores late firing of telomeric origins in cells devoid of Yku70/80 [42–44,46]. To explore the possibility that Tel1-dependent activation of telomeric origins in early S might influence the response to NAM-induced replicative stress in *yku70Δ* mutants, we first evaluated the impact of Tel1 on S phase progression in *yku70Δ* cells. Strikingly, NAM-treated *yku70Δ* mutants accumulated in early-mid S in a Tel1-dependent manner, indicating that Tel1 influences global dynamics of DNA replication in *yku70Δ* cells experiencing replicative stress (Fig 3B). Pre-senescent telomerase mutants (*est1Δ* and *est2Δ*) also presented NAM-induced S phase progression defects which became worse over cell generations (Fig 3C), strongly suggesting a link between progressive shortening of telomeres and compromised DNA replication. Interestingly, we did not detect significant increase in the frequency of Rad52-YFP and Rfa1-YFP foci in *yku70Δ* cells exposed to NAM compared to WT, indicating that replication defects in cells with short telomeres are unlikely to result from elevated induction of DNA lesions in these conditions (Fig 3D and 3E). We note that replication proceeds slowly in telomeric regions even in the absence of exogenous DNA damage, presumably because non-histone protein complexes impede replication fork (RF) progression at these loci [80]. Indeed, cells devoid of the Rrm3 helicase, which promotes DNA replication across genomic regions harboring chromatin-bound protein complexes, display a 10-fold increases in the number of stalled RFs at telomeres [80]. We found that deletion of *RRM3* caused synthetic MMS sensitivity when combined with *yku70Δ* (Fig 3F), suggesting that an abnormal abundance of stalled RFs at telomeres might contribute to the phenotypes of cells lacking Yku70/80.

Our data indicate genome-wide reduction in replication progression in NAM-treated *yku70Δ* cells vs WT (Fig 3C), which cannot only reflect abnormal RF progression at telomeric/subtelomeric regions since they represent a minor proportion of a cell’s total DNA. Instead, the Tel1-dependent NAM sensitivity of *yku70Δ* mutants suggests that abnormal activation of telomeric origins in early S influence DNA replication dynamics genome-wide. Elevated origin activation at repetitive loci harboring replication origins, e.g., rDNA repeats in *sir2Δ* mutants, diminishes origin activity at unlinked loci by titrating replication initiation factors that are in limiting abundance [81,82]. Lack of *YKU70* causes misregulation of a significant number of origins: yeast cells possess at least one origin per telomere (32 per haploid cell), and origin activation repression extends up to 40 kb inward from chromosome ends [44]. We hypothesized that origin activation in early S at telomeric regions, combined with that occurring at canonical early replicating loci, might i) generate an overwhelming number of stalled RFs in early S in response to NAM-induced replicative stress, leading to ii) sequestration of replication factors. This might compromise activation of origins throughout chromosomes and overall replication progression in mid/late S, and prevent rescue of stalled RF by converging forks. We first tested whether increasing the number of replication origins competing for limiting pools of factors sensitizes otherwise WT cells to replicative stress. Consistently, cells harboring 200–400 copies of a YEPFAT7.5 2μ plasmid [83], which depends on endogenous DNA replication factors for its propagation, were noticeably more sensitive to MMS and NAM than WT cells (S7A and S7B Fig). Conversely, we reasoned that increasing the availability of limiting DNA replication initiation factors might rescue replicative stress-induced growth defects in cells lacking Yku70/80. Cdc45, Sld3 and Sld7 (45/3/7) are required to trigger activation of licensed replication origins; moreover, these factors are limiting in abundance and their overexpression rescues genome-wide anomalies in DNA replication dynamics caused by early S activation of rDNA origins in *sir2Δ* mutants [81,84]. We found that 45/3/7 overexpression partially rescued growth of *yku70Δ* mutants in response to MMS or NAM (Fig 3G and 3H), as well as the strong synthetic sensitivity of *rad52Δ yku70Δ* mutants to MMS (Fig 3I). These results suggest that increasing the availability of limiting replication factors can mitigate DNA replication defects caused by short telomeres in *yku70Δ* cells.

### H4K16ac influences the sensitivity to replicative stress of cells with short telomeres by modulating DNA damage-induced signaling

**Fig 4:**
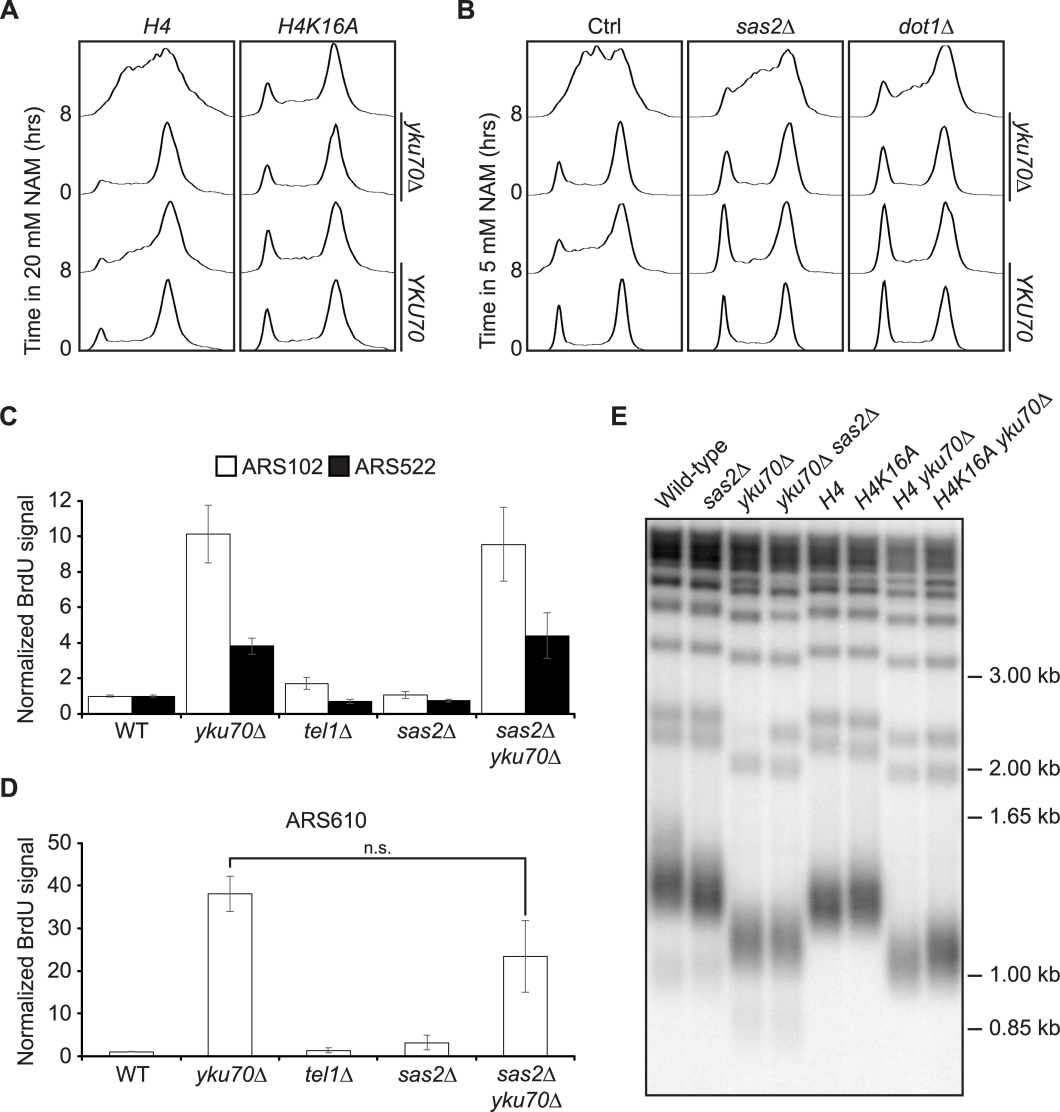
Preventing H4K16 acetylation rescues S phase progression defects without modulating telomere length or telomeric origin activity. (A-B) Reducing H4K16ac and H3K79me levels rescues S phase progression defects of *yku70Δ* mutants. Asynchronous cells were incubated in YPD for 8 hours at 30°C in the presence of 20 mM (A) or 5mM (B) NAM. Samples were taken at indicated time for flow cytometry-based DNA content analysis. (C-D) *SAS2* deletion does not prevent early activation of telomeric origins in *yku70Δ* cells. Cells were synchronized in G1 and released toward S phase in the presence of 200 mM hydroxyurea. 30 minutes before release, 400 ug/mL BrdU was added to cultures. Sonicated BrdU-labelled DNA was immunoprecipitated and recovered material from telomeric/subtelomeric origins was quantified by qPCR as described in materials and methods. Error bars: Standard error of the mean. (E) H4K16ac levels do not significantly influence telomere length. Telomere length was analysed by southern blotting using a probe that recognizes the telomeric TG_1-3_ repeats.

As was the case for *tel1Δ*, the *H4K16A* mutation rescued NAM-induced S-phase progression defects in *yku70Δ* cells (Fig 4A and 4B). The effect of *sas2Δ* was partial, which may reflect the fact that this mutation does not completely abolish H4K16ac, in contrast to *H4K16A* [22]. We first explored the possibility that mutations abolishing H4K16ac might suppress replication-associated phenotypes of *yku70Δ* mutants by reversing early activation of telomeric and subtelomeric origins. We synchronized cells in G1 using alpha factor and released them in early S in the presence of hydroxyurea (HU) and BrdU for 90 minutes, followed by BrdU immunoprecipitation and quantitative PCR to monitor dNTP incorporation at origins. We note that the amplitude of signals obtained by this technique has been shown to correlate well with origin activation/efficiency in early S phase [85–88]. We examined BrdU incorporation at two telomeric origins, ARS102 and ARS610, and one sub-telomeric origin, ARS522. As expected, these origins were more active when telomeres are shortened in the *yku70Δ* mutant, but not in *tel1Δ* cells (Fig 4C and 4D). We found that *sas2Δ* did not significantly influence the activity of telomeric/subtelomeric replication origins, alone or when combined with *YKU70* deletion (Fig 4C and 4D). Importantly, neither *sas2Δ* or *H4K16A* significantly modulated telomere length (Fig 4E), indicating that H4K16ac impedes S phase progression of cells with short telomeres via other mechanisms.

**Fig 5:**
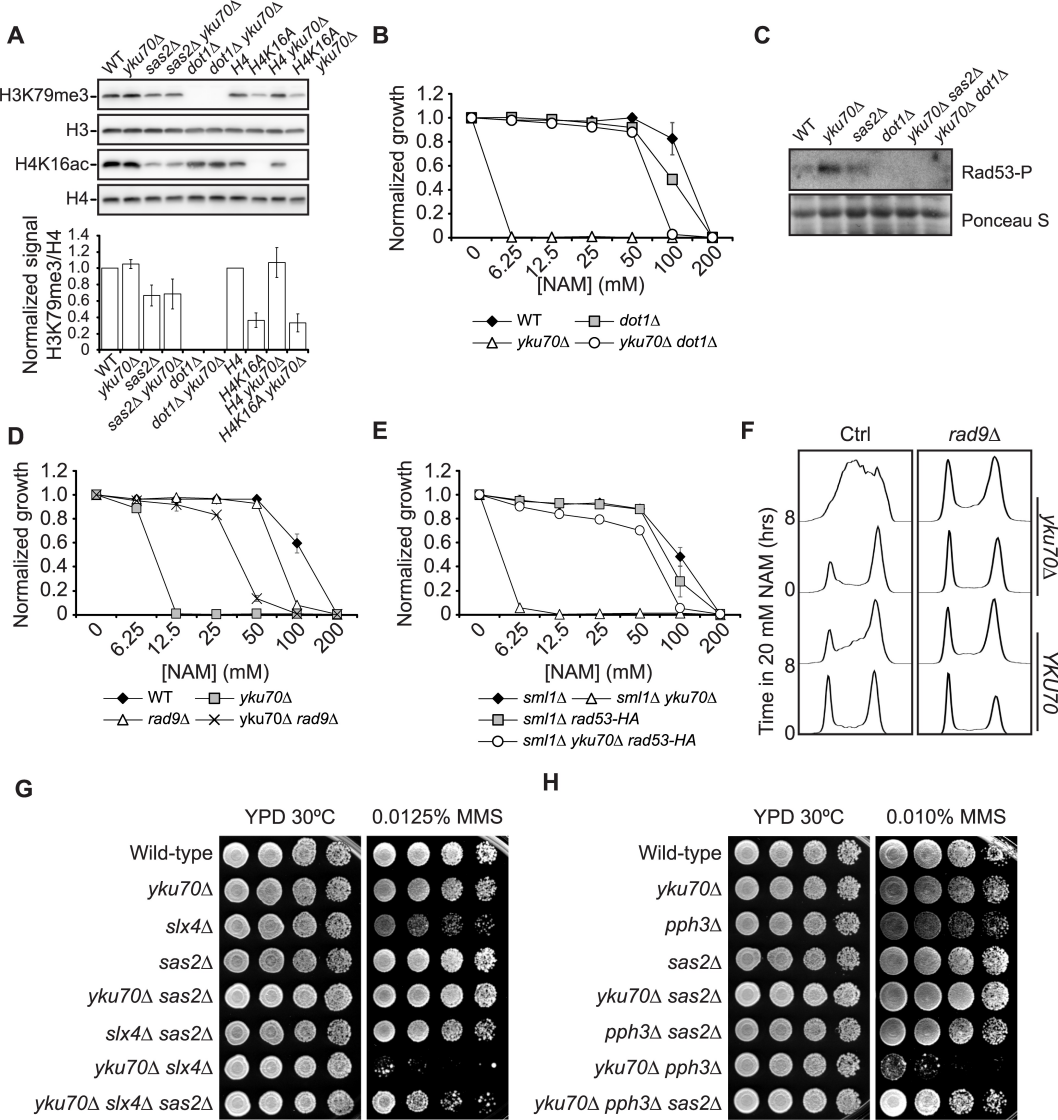
Elevated DDR signalling is deleterious for growth of *yku70Δ* cells upon NAM- and MMS-induced replicative stress. (A) Mutations that abolish H4K16ac cause a reduction in H3K79me3 levels. Protein samples from asynchronous cells were immunoblotted with indicated antibodies. Bar graph represents the ratio of H3K79me3 onto H4 signals as quantified by densitometry. Error bars: standard error of the mean (B) Deletion of *DOT1* rescues growth of *yku70Δ* mutants in NAM (C) *sas2Δ* and *dot1Δ* mutations reduce Rad53 activation in *yku70Δ* cells. Cells were exposed to 20 mM NAM for 8 hours at 30°C and samples were taken for Rad53 *in situ* autophosphorylation assays (see materials and methods for details). (D-E) *rad9Δ* (D) and *RAD53-HA* (E), which limit DDR signalling in response to replicative stress, rescue growth of *yku70Δ* mutants in NAM. Growth assay in 96-well plates (see materials and methods). Error bars: standard deviation. (F) Deletion of *RAD9* rescues S phase progression defects of *yku70Δ* cells exposed to NAM. Asynchronous cells were incubated in YPD for 8 hours at 30°C in the presence of 20 mM NAM. Samples were taken at indicated time for flow cytometry-based DNA content analysis. (G-H) *yku70Δ* display synthetic sensitivity to MMS when combined with *slx4Δ* (G) and *pph3Δ* (H) mutants.

We previously showed that mutations which prevent H4K16ac cause a reduction in tri-methylated H3K79 (H3K79me3) levels [22], an effect that we also observed in *yku70Δ* mutants (Fig 5A). Interestingly, we found that abolishing H3K79 methylation via deletion of the histone methyltransferase-encoding gene *DOT1* (Fig 5A) significantly rescued growth and S phase progression defects in *yku70Δ* cells exposed to NAM (Fig 4B, Fig 5B). This suggests that H4K16ac might influence the NAM sensitivity of *yku70Δ* cells at least in part by modulating H3K79 methylation levels. While the biological consequences and molecular mechanisms of co-reduction in H4K16ac and H3K79me3 are uncharacterized, we originally speculated that modulation of H4K16ac might influence the DNA damage response (DDR) by influencing the recruitment of the H3K79me3-binding DDR protein Rad9 to damaged chromatin and subsequent Rad53 activation [24–27]. We found that upon exposure to NAM, *yku70Δ* cells displayed increased Rad53 activation, which was strongly reduced by mutating either *SAS2* or *DOT1* (Fig 5C). Directly limiting the amplitude of DDR signaling, via *RAD9* mutation or expression of a hypomorphic *rad53-HA* allele [33], rescued the growth of *yku70Δ* mutants in NAM (Fig 5D and 5E). *rad9Δ* also rescued DNA replication progression in *yku70Δ* cells upon NAM exposure (Fig 5F). Conversely, deletion of either *PPH3* or *SLX4*, which cripples cellular pathways that act to limit Rad53 activation upon replicative stress [30,31], caused synthetic sensitivity to MMS when combined with *yku70Δ*, an effect which was found to depend on the H4K16ac acetyltransferase Sas2 (Fig 5G and 5H). Taken together, our results indicate that i) co-dependent H4K16ac and H3K79me3 promote DDR signalling in *yku70Δ* mutants, and ii) elevated Rad53 activity contributes significantly to the sensitivity of cells lacking Yku70 to MMS and NAM-induced replicative stress.

### H4K16ac and H3K79me control translesion synthesis usage in *yku70Δ* mutants

**Fig 6:**
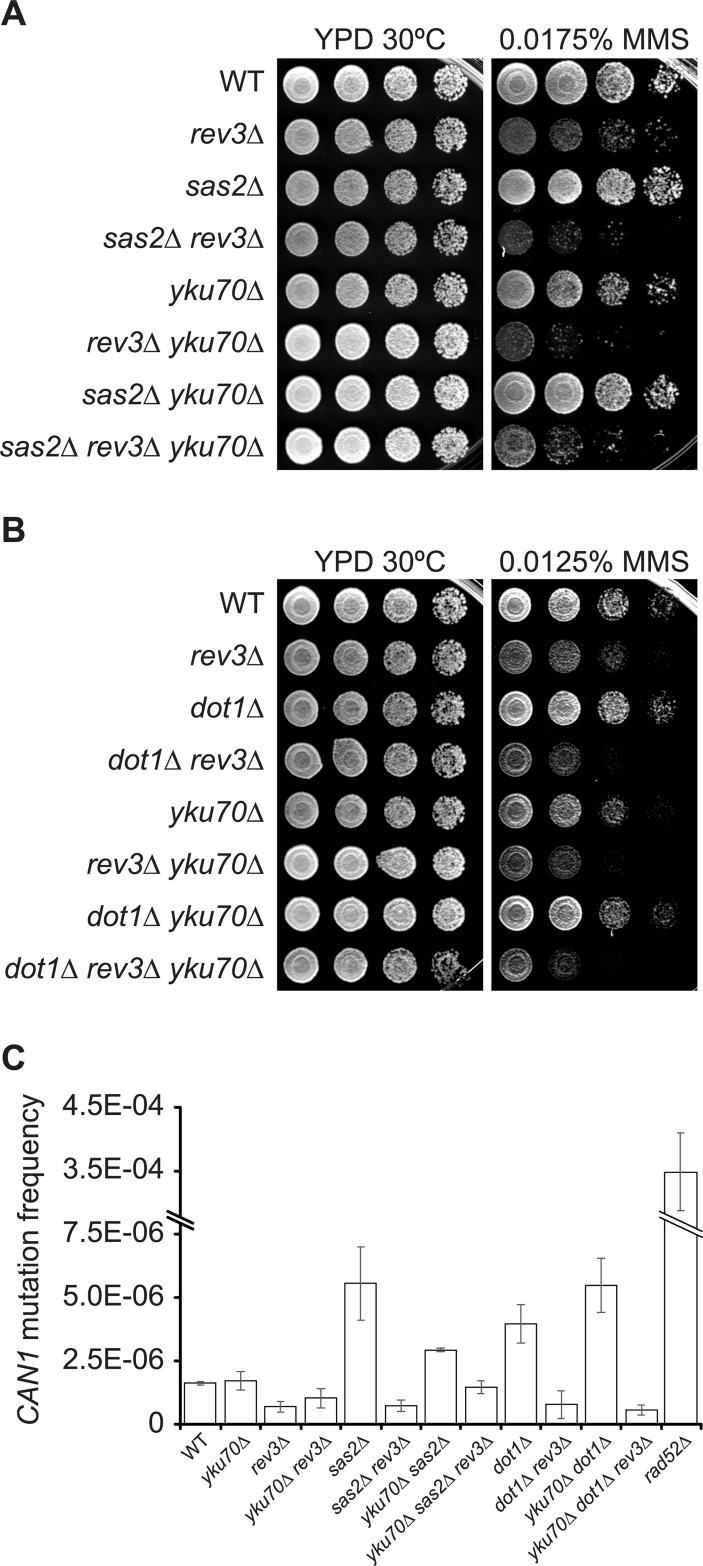
H4K16ac or H3K79me levels influence translesion synthesis in *yku70Δ* cells exposed to MMS-induced replicative stress. (A-B) *sas2Δ* or *dot1Δ* rescue the growth of *yku70Δ* cells in MMS in a Rev3-dependent manner. (C) *sas2Δ* increases MMS-induced *CAN1* mutation frequency in *yku70Δ* or otherwise wild-type cells in a Rev3-dependent manner. Mutation frequency was assessed as described in materials and methods. Error bars: standard error of the mean.

Rad53 activation is known to inhibit DNA replication origin firing, thereby delaying S phase progression upon replicative stress [89,90]. This effect can be bypassed by preventing Rad53-dependent phosphorylation of Dbf4 and Sld3, which are two key proteins of the origin activation cascade [90]. We found that *yku70Δ* yeast strains expressing non-phosphorylable alleles of Dbf4 and Sld3 are as sensitive to NAM as control strains (S8 Fig), suggesting that other consequences of Rad53 hyperactivation influence the ability of cells lacking Yku70/80 to proliferate in response to replicative stress. Limiting the activation of Rad53, by deleting *DOT1* or expressing a hypomorphic *rad53-HA* allele, increases resistance to MMS-induced replicative stress by elevating lesion bypass via the translesion synthesis (TLS) pathway [33,34]. Our data indicate that the sensitivity to MMS of cells lacking Yku70 complex is exacerbated by mutating *REV3*, which encodes the catalytic subunit of TLS polymerase zeta required for the bypass of MMS-induced lesions (Fig 6A and 6B). In addition, we found that the rescue of the MMS sensitivity of *yku70Δ* by deletion of *SAS2* or *DOT1* depends on Rev3 (Fig 6A and 6B). TLS is intrinsically error-prone, and elevated usage on this pathway increases mutagenesis [91]. Concordantly, deletion of *SAS2* or *DOT1* in either WT or *yku70Δ* cells led to a statistically significant increase in MMS-induced *CAN1* mutation frequency, an effect which was reverted by *rev3Δ* (Fig 6C, p-value < 0.05). Together, these results suggest that H4K16ac and H3K79me influence TLS-dependent bypass of replication blocking DNA lesions in cells lacking Yku70/80 by modulating DDR signalling.

## Discussion

In the current study, we investigated the molecular basis of the sensitivity of cells lacking Yku70/80 to the pan-sirtuin inhibitor NAM. Our results reveal a novel interplay between telomere length and sirtuin-mediated regulation of chromatin structure in protecting cells against DNA replication stress. Indeed, we provide compelling evidence that in the absence of Yku70/80, telomere shortening, but not NHEJ deficiency nor other telomere-related defects, diminishes the ability of yeast cells to respond to replicative stress. Notably, we found that telomerase (*est1/2Δ*) mutants phenocopied *yku70Δ* both in terms of NAM-induced growth defects and S phase accumulation, and that manipulating telomere length via inducible Yku70 degradation or expression of a Cdc13-Est1 fusion modulates the sensitivity of cells to NAM. We note that sudden telomere shortening occurs naturally *in vivo* in WT yeast and is thought to result from replication fork collapse in telomeric tracts [92]. However, since such telomere shortening events are relatively infrequent and are expected to involve only one or a few telomeres in any given cell, the impact on DNA replication stress responses as described here is expected to be minor. This is in contrast with the situation for cells exhibiting uniform reduction in the length of all telomeres, such as telomerase and *yku70Δ/yku80Δ* mutants. Overall, our results are consistent with the fact that senescence caused by lack of telomerase activity is associated with induction of classical markers of the DNA damage response [93,94], and raise the interesting possibility that compromised responses to replicative stress might be a general phenomenon arising in mutants with short telomeres.

An important exception to the above involves the *tel1ΔΔ* mutation which, despite rendering telomeres extremely short, does not sensitize cells to NAM. Rather, we found that deletion of *TEL1* rescued NAM-induced growth inhibition and DNA replication defects in *yku70Δ* cells. Previous reports established that telomere shortening caused by deletion of *YKU70/80* provokes Tel1-dependent firing of telomeric origins in early S phase, as opposed to late S in WT cells [43–46]. Since RFs progress slowly within telomeric and subtelomeric regions [70,80,95], it is plausible that activation of telomeric origins in early S in *yku70Δ* cells, but not in *yku70Δ tel1Δ* mutants, generates an overwhelming number of stalled RFs over a short period in the presence of replicative stress-inducing drugs. In addition, we provide evidence suggesting that RF stalling at telomeric regions in early S may compromise genome-wide replication dynamics by causing sequestration, and eventual exhaustion, of limiting replication factors, which in turn is expected to negatively impact further activation of origins later in S. We propose that such reduction in RF initiation events might prevent rescue of stalled RFs by converging forks in mid/late S, thereby contributing to the sensitivity of *yku70Δ* cells to replicative stress. We note however that while the above-described models rationalize our observations regarding the impact of Tel1 on the sensitivity of *yku70Δ* cells to NAM, we cannot exclude that consequences of telomere shortening other than misregulation of telomeric origins might also be implicated.

Consistent with the notion that cells with short telomeres exhibit an elevated number of stalled RFs during replicative stress, we found that the DDR kinase Rad53 is strongly activated upon NAM exposure in *yku70Δ* cells. Our data further indicate that such hyperactive DDR signalling contributes to the phenotypes of these mutants; indeed, we found that i) *yku70Δ* causes synthetic sensitivity to MMS when combined with either *slx4Δ* or *pph3Δ*, which are both known to limit Rad53 activity in response to DNA replication impediments [30–32], and ii) mutations that cripple Rad53 activation rescue the NAM sensitivity of *yku70Δ* cells. While the consequences of Rad53 hyperactivation during replicative stress are incompletely characterized, our published data and those of others [33,34] suggest that restricting DDR signalling improves cell survival and replication progression in response to genotoxins at least in part by promoting DNA damage tolerance via TLS. Overall, the results presented here are consistent with the above, and highlight the importance of mechanisms that dampen DDR signalling in promoting the survival of cells with short telomeres upon DNA replication stress.

In contrast to most other mutations causing NAM sensitivity that we analysed so far [35], deletion of *YKU70* does not cause synthetic lethality when combined with *hst3Δ hst4Δ*, implying that other sirtuins are essential for survival of cells presenting short telomeres. Indeed, our genetic data support the notion that the redundant ability of Sir2 and Hst1 to deacetylate H4K16ac promotes resistance to replicative stress in cells lacking Yku70/80, whereas Hst3/4-dependent removal of H3K56ac mainly acts to limit the generation of endogenous DNA damage in this context. We note that even though the identity of the DNA lesions generated by constitutive H3K56ac is unknown, our results showing that the ability of Yku70/80 to promote NHEJ is not necessary for NAM resistance suggest that the predominant lesions caused by hyperacetylated H3K56 are unlikely to be DSBs. This is also consistent with the sensitivity of *yku70Δ* mutant to MMS, which produces few, if any, DSBs in yeast [55]. While other Sir2/Hst1 targets might also contribute to this phenomenon, abolishing H4K16ac rescued the NAM, MMS, and temperature sensitivity of *yku70Δ* cells, indicating that this histone modification is a critical determinant of the phenotypes of these mutants. We note that the SIR complex was previously shown to suppress origin firing at telomeres [41] which, combined with our results, raised the possibility that this effect might depend on H4K16ac levels. However contrary to this idea, our data clearly indicate that reducing H4K16ac by deletion of the *SAS2* acetyltransferase does not impact origin activity at short telomeres (Fig 4), consistent with a prior report indicating that telomeric origins in *yku70Δ* mutants are activated in early S independently of histone tail acetylation [44].

Our data highlight a novel role for H4K16ac regulation in limiting the activation of Rad53 upon replicative stress in *yku70Δ* mutants, presumably by modulating H3K79 trimethylation levels and Rad9 activity/recruitment to chromatin. This is noteworthy since H4K16ac is very abundant and is removed by sirtuins only at specific transcriptionally silent genomic loci, e.g., mating loci (HMR and HML), as well as at telomeric regions [29,57,96]. These regions present intrinsic impediments to DNA replication fork progression, often in the form of chromatin-bound protein complexes [80,97–99]. In view of this, our results raise the intriguing possibility that cells may have evolved mechanisms to limit H4K16ac levels in these genomic regions in part to mitigate the deleterious consequences of unchecked DDR signalling arising from frequent RF stalling. We also note that senescent yeast cells have been shown to manifest reduced levels of Sir2, and consequently exhibit elevated H4K16ac at telomeric regions [100]. Moreover, *SAS2* deletion was demonstrated to extend replicative life span [100]. It is tempting to speculate that misregulation of H4K16ac might contribute to certain phenotypes of senescent cells, e.g., elevated DNA damage, by promoting intense DDR signaling in response to spontaneous replicative stress arising at loci that are intrinsically difficult to replicate, including telomeric regions [101–103].

## Materials and methods

### Yeast strains and growth conditions

Experiments were performed using standard yeast growth conditions. Yeast strains used in this study are listed in Table 1. To avoid frequent emergence of spontaneous suppressor mutations in cells with constitutive H3K56 hyperacetylation, *hst3Δ hst4Δ* strains used in this study were propagated with a *URA3*-harboring centromeric plasmid encoding Hst3. To evaluate the phenotypes caused by *hst3Δ hst4Δ*, cells were plated on 5-Fluoroorotic Acid (5-FOA)-containing medium immediately before experiments to select cells that spontaneously lost the plasmid, or during the experiment (spot assays on 5-FOA-containing plates). For experiments involving telomerase mutants (*est1Δ* or *est2Δ*), fresh haploid clones were obtained from tetrad dissection of heterozygous diploids to ensure that cells were not undergoing senescence during experiments. For spot assays, cells were grown to saturation in YEP with 2% glucose or 2% raffinose in a 96-well plate. Five-fold serial dilutions of these cultures with identical OD were then plated on indicated media and allowed to grow for 2 to 5 days. Growth assays in NAM were done as previously described [35]. Cells were diluted to OD_600_ 0.0005 in 100 μL of YPD with increasing NAM concentrations in a 96-well plate. OD_630_ were acquired using a BioTek EL800 plate reader, and growth of each strain was normalized relative to an untreated control well. For doubling time assessments, cells were diluted to OD_600_ 0.01 in 100 μL of YPD in a 96-well plate and incubated at 30°C in a BioTek EL808 plate reader for 48h. Every 30 minutes, plates were shaken for 30 seconds and OD_630_ readings were acquired. Doubling times were derived from exponential regression of the resulting growth curve.

**Table 1:** Strains used in this study.

Strain	Genotype	Reference
BY4741	BY4741 *MATa ura3Δ0 leu2Δ0 his3Δ1*	[104]
BY4743	BY4743 *MATa/α his3Δ*1/*his3Δ1 leu2Δ0*/*leu2Δ0 LYS2*/*lys2Δ0 met15Δ0*/*MET15 ura3Δ0*/*ura3Δ0*	[104]
ASY4249	BY4741 *MATa hst3Δ*::*HPHMX hst4Δ*::*NATMX [pHST3 URA3]*	[35]
ASY5043	BY4741 *MATa hst3Δ*::*HPHMX hst4Δ*::*NATMX yku70Δ*::*KanMX [pHST3 URA3]*	This study
ASY1767	BY4741 *MATa yku70Δ*::*KanMX*	[35]
ASY4526	BY4741 *MATa sas2Δ*::*KanMX*	This study
ASY4836	BY4741 *MATa sas2Δ*::*URA3MX yku70Δ*::*KanMX*	This study
ASY3111	YBL574 *hht1-hhf1Δ*::*LEU2 hht2-hhf2Δ*::*HIS3* [*pCEN TRP1 HHT1-HHF1*]	[105]
ASY3113	YBL574 *hht1-hhf1Δ*::*LEU2 hht2-hhf2Δ*::*HIS3* [*pCEN TRP1 HHT-hhf1K16A*]	[105]
ERY3398	YBL574 *hht1-hhf1Δ*::*LEU2 hht2-hhf2Δ*::*HIS3* [*pCEN TRP1 HHT1-HHF1*] *yku70Δ*::*KanMX*	This study
ERY3400	YBL574 *hht1-hhf1Δ*::*LEU2 hht2-hhf2Δ*::*HIS3* [*pCEN TRP1 HHT-hhf1K16A*] *yku70Δ*::*KanMX*	This study
ASY4460	BY4741 *MATa hst3Δ*::*HPHMX hst4Δ*::*NATMX sir3Δ*::*KanMX [pHST3 URA3]*	This study
ASY4282	BY4741 *MATa hst3Δ*::*HPHMX hst4Δ*::*NATMX sir4Δ*::*KanMX [pHST3 URA3]*	This study
ASY4528	BY4741 *MATa hst3Δ*::*HPHMX hst4Δ*::*NATMX sir3Δ*::*HIS3MX yku70Δ*::*KanMX* *[pHST3 URA3]*	This study
ASY4516	BY4741 *MATa hst3Δ*::*HPHMX hst4Δ*::*NATMX sir4Δ*::*HIS3MX yku70Δ*::*KanMX* *[pHST3 URA3]*	This study
ASY4868	BY4741 *MATa hst3Δ*::*HPHMX hst4Δ*::*NATMX sir3Δ*::*HIS3MX sir4Δ*::*KanMX* *[pHST3 URA3]*	This study
ASY5108	BY4741 *MATa hst3Δ*::*HPHMX hst4Δ*::*NATMX hst1Δ*::*HIS3MX [pHST3 URA3]*	This study
ASY5110	BY4741 *MATa hst3*Δ::*HPHMX hst4Δ*::*NATMX yku70Δ*::*KanMX hst1Δ*::*HIS3MX* *[pHST3 URA3]*	This study
ASY5118	BY4741 *MATa hst3Δ*::*HPHMX hst4Δ*::*NATMX sum1Δ*::*HIS3MX [pHST3 URA3]*	This study
ASY5121	BY4741 *MATa hst3Δ*::*HPHMX hst4Δ*::*NATMX yku70Δ*::*KanMX sum1Δ*::*HIS3MX* *[pHST3 URA3]*	This study
ASY4038	BY4741 *MATa hst1Δ*::*KanMX*	This study
ASY4040	BY4741 *MATa sir2Δ*::*KanMX*	This study
ASY3975	BY4741 *MATa hst1Δ*::*KanMX yku70Δ*::*HPHMX*	This study
ASY3727	BY4741 *MATa sir2Δ*::*KanMX yku70Δ*::*HPHMX*	This study
ASY5130	BY4741 *MATa sir2Δ*::*KanMX hst1Δ*::*HIS3MX*	This study
ASY5132	BY4741 *MATa sir2Δ*::*KanMX hst1Δ*::*HIS3MX yku70Δ*::*HPHMX*	This study
HWY289	BY4741 *MATa fob1Δ*::*KanMX*	This study
ERY4186	BY4741 *MATa fob1Δ*::*KanMX yku70Δ*::*HPHMX*	This study
ASY5113	BY4741 *MATa sum1Δ*::*HIS3MX*	This study
ASY5116	BY4741 *MATa sum1Δ*::*HIS3MX yku70Δ*::*KanMX*	This study
ASY5147	BY4741 *MATa sum1Δ*::*URA3MX yku70Δ*::*HPHMX sir2Δ*::*KanMX hst1Δ*::*HIS3MX*	This study
ASY4794	YBL574 *hht1-hhf1Δ*::*LEU2 hht2-hhf2Δ*::*HIS3* [*pCEN TRP1 HHT1-HHF1*] *sum1Δ*::*HPHMX*	This study
ASY4797	YBL574 *hht1-hhf1Δ*::*LEU2 hht2-hhf2Δ*::*HIS3* [*pCEN TRP1 HHT-hhf1K16A*] *sum1Δ*::*HPHMX*	This study
ASY4800	YBL574 *hht1-hhf1Δ*::*LEU2 hht2-hhf2Δ*::*HIS3* [*pCEN TRP1 HHT1-HHF1*] *yku70Δ*::*KanMX sum1Δ*::*HPHMX*	This study
ASY4801	YBL574 *hht1-hhf1Δ*::*LEU2 hht2-hhf2Δ*::*HIS3* [*pCEN TRP1 HHT-hhf1K16A*] *yku70Δ*::*KanMX sum1Δ*::*HPHMX*	This study
ASY2229	BY4741 *MATa dnl4Δ*::*KanMX*	This study
ASY2230	BY4741 *MATa nej1Δ*::*KanMX*	This study
ASY2231	BY4741 *MATa lif1Δ*::*KanMX*	This study
ASY1762	BY4741 *MATa yku80Δ*::*KanMX*	This study
ASY4104	BY4743 *MATa/α est1Δ*::*KanMX/EST1*	This study
ASY4105	BY4743 *MATa/α est2Δ*::*KanMX/EST2*	This study
ASY3689	BY4741 *MATa tel1Δ*::*KanMX*	This study
ASY3715	BY4741 *MATa tel1Δ*::*KanMX yku70Δ*::*HPHMX*	This study
HWY2678	BY4741 *MATa TIR1-Myc*::*URA3MX*	This study
ASY4083	BY4741 *MATa TIR1-Myc*::*URA3MX YKU70-6FLAG-AID*::*HPHMX*	This study
YAB471	W303 *MATa cdc13Δ*::*NATMX* pVL438	[51,76]
YAB718	W303 *MATa yku80Δ*::*HPHMX cdc13Δ*::*NATMX* pVL438	[51,76]
W5094-1C	W303 *ADE2 RAD52*-*YFP RAD5*	[106]
HWY2841	W303 *ADE2 RAD52*-*YFP RAD5 yku70Δ*::*KanMX*	This study
1962	W303 *MATa his3*::*GAL-MHT*	[81,84]
1964	W303 *MATa his3*::*GAL-SLD3/SLD7/CDC45* (2 copies)	[81,84]
ASY4876	W303 *MATa his3*::*GAL-MHT yku70Δ*::*KanMX*	This study
ASY4882	W303 *MATa his3*::*GAL-SLD3/SLD7/CDC45* (2 copies) *yku70Δ*::*KanMX*	This study
ASY5181	W303 *MATa his3*::*GAL-MHT rad52Δ*::*HPHMX*	This study
ASY5185	W303 *MATa his3*::*GAL-SLD3/SLD7/CDC45* (2 copies) *rad52Δ*::*HPHMX*	This study
ASY5268	W303 *MATa his3*::*GAL-MHT rad52Δ*::*HPHMX yku70Δ*::*KanMX*	This study
ASY5272	W303 *MATa his3*::*GAL-SLD3/SLD7/CDC45* (2 copies) *rad52Δ*::*HPHMX yku70Δ*::*KanMX*	This study
HWY99	BY4741 *MATα LEU2*	This study
ASY5345	BY4741 *MATa p[YEPFAT7*.*5 leu2d URA3]*	This study
HWY534	BY4741 *his3*::*p403-BrdU-Inc*	[106]
ASY5097	BY4741 *his3*::*p403-BrdU-Inc yku70Δ*::*URA3MX*	This study
ASY5320	BY4741 *his3*::*p403-BrdU-Inc tel1Δ*::*KanMX*	This study
ASY5331	BY4741 *his3*::*p403-BrdU-Inc sas2Δ*::*KanMX*	This study
ASY5334	BY4741 *his3*::*p403-BrdU-Inc sas2Δ*::*KanMX yku70Δ*::*URA3MX*	This study
HWY3892	BY4741 *MATa dot1Δ*::*KanMX*	[35]
ERY3391	BY4741 *MATa yku70Δ*::*KanMX dot1Δ*::*URA3*	This study
EHY1120	BY4741 *MATa rad9Δ*::*KanMX*	[35]
ASY3681	BY4741 *MATa sml1Δ*::*KanMX*	[35]
ASY4467	BY4741 *MATa yku70Δ*::*KanMX rad9Δ*::*URA3*	This study
ASY4853	BY4741 *MATa yku70Δ*::*URA3 sml1Δ*::*KanMX*	This study
ASY5603	BY4741 *MATa sml1Δ*::*KanMX rad53-HA*::*HIS3MX*	This study
ASY5605	BY4741 *MATa yku70Δ*::*URA3 sml1Δ*::*KanMX rad53-HA*::*HIS3MX*	This study
HWY1608	BY4741 *MATa slx4Δ*::*KanMX*	[35]
ASY1835	BY4741 *MATa pph3Δ*::*HPHMX*	[35]
ASY4839	BY4741 *MATa yku70Δ*::*KanMX slx4Δ*::*URA3*	This study
ASY4842	BY4741 *MATa yku70Δ*::*KanMX pph3Δ*::*URA3*	This study
ASY5592	BY4741 *MATa slx4Δ*::*KanMX sas2Δ*::*HIS3MX*	This study
ASY5595	BY4741 *MATa pph3Δ*::*KanMX sas2Δ*::*HIS3MX*	This study
ASY5606	BY4741 *MATa yku70Δ*::*KanMX slx4Δ*::*URA3 sas2Δ*::*HIS3MX*	This study
ASY5609	BY4741 *MATa yku70Δ*::*KanMX pph3Δ*::*URA3 sas2Δ*::*HIS3MX*	This study
HWY3893	BY4741 *MATa rev3Δ*::*KanMX*	[35]
ASY5597	BY4741 *MATa sas2Δ*::*KanMX rev3Δ*::*HIS3MX*	This study
ASY4832	BY4741 *MATa yku70Δ*::*KanMX rev3Δ*::*URA3*	This study
ASY5600	BY4741 *MATa yku70Δ*::*KanMX sas2Δ*::*URA3 rev3Δ*::*HIS3MX*	This study
ASY4856	BY4741 *MATa dot1Δ*::*KanMX rev3Δ*::*HPHMX*	This study
ASY4857	BY4741 *MATa dot1Δ*::*URA3 yku70*Δ::*KanMX rev3Δ*::*HPHMX*	This study
ASY1873	BY4741 *MATa rad52Δ*::*KanMX*	This study
HWY329	BY4741 *MATa rrm3Δ*::*KanMX*	This study
ASY4829	BY4741 *MATa yku70Δ*::*KanMX rrm3Δ*::*URA3*	This study
Y2573	W303 MATa *dbf4Δ*::*TRP1 his3*::*PDBF4-dbf4–4A*::*HIS3 sld3–38A-10his-13MYC*::*KanMX4*	[90]
ERY3859	W303 MATa *dbf4Δ*::*TRP1 his3*::*PDBF4-dbf4–4A*::*HIS3 sld3–38A-10his-13MYC*::*KanMX4* *yku70Δ*::*HPHMX*	This study
ASY4217	BY4741 *MATa elg1Δ*::*KanMX*	This study
ASY4218	BY4741 *MATa elg1Δ*::*KanMX yku70Δ*::*HPHMX*	This study
ASY3223	YBL574 *hht1-hhf1Δ*::*LEU2 hht2-hhf2Δ*::*HIS3* [*pCEN TRP1 hht1K56A-HHF1*]	[105]
ASY5612	YBL574 *hht1-hhf1Δ*::*LEU2 hht2-hhf2Δ*::*HIS3* [*pCEN TRP1 hht1K56A-HHF1*] *yku70Δ*::*KanMX*	This study

### Telomere southern blot

Monitoring of telomere length by southern blotting was performed as described [107]. Briefly, genomic DNA was digested with XhoI (New England Biolabs) and run on a 1.2% agarose gel for 17 hrs in 1x TBE buffer. Telomeric repeats were detected with a TG_1-3_ probe kindly provided by Dr Raymund Wellinger (Université de Sherbrooke).

### Immunoblotting

Proteins were extracted from samples by alkaline cell lysis [108] and run on 10% or 15% acrylamidegels to resolve Yku70and histones respectively. Flag epitope was detected using an anti-Flag-M2 antibody (Sigma), histones modifications were detected using anti-H3K79me3 (Abcam, AB2621) and anti-H4K16ac (EMD Millipore, 07–329) antibodies. Antibodies against histone H3 (AV100) and histone H4 (AV95) were kindly provided by Dr Alain Verreault (Université de Montréal).

### DNA content analysis by flow cytometry

Cells were fixed in 70% ethanol, sonicated, treated with 0.4 ug/mL RNAse A in 50mM Tris-HCl pH 7.5 for 3 hours at 42°C followed by treatment with 1mg/mL Proteinase K in 50mM Tris-HCl pH 7.5 for 30 minutes at 50°C. DNA content was assessed by Sytox Green (Invitrogen) staining as previously described [109]. DNA content analysis was performed on a FACS Calibur flow cytometer equipped with Cell Quest software. Graphs were produced using FlowJo 7.6.5 (FlowJo, LLC).

### Fluorescence microscopy

Cells expressing Rad52-YFP or Rfa1-YFP were fixed with formaldehyde as previously described [106,110] and stained with DAPI. Fluorescence was examined with a DeltaVision microscope equipped with SoftWorx version 6.2.0 software (GE Healthcare). Images were examined using a custom MATLAB script (version R2017a; MathWorks) to extract the number of cells with Rfa1-YFP or Rad52-YFP foci. Briefly, a mask was created based on DAPI signals to identify cell nuclei and count the number of cells within an image. A second mask was created with the YFP channel to mark foci by finding spots with elevated YFP fluorescence compared to surrounding regions. Nuclei with at least one focus were listed as cells with Rfa1-YFP foci.

### *in situ* Rad53 autophosphorylation assays

Protein samples were prepared by trichloroacetic acid/glass beads lysis, separated on 10% acrylamide gels and transferred to a PVDF membrane. Autophosphorylation assays were carried out as previously described [111].

### Auxin-induced degradation

Cells were maintained in logarithmic phase for the indicated number of days (see Fig 2) by dilution in fresh YPD ± 2 mM Auxin (3-indoleacetic acid, Sigma). For each time point, a growth assay was performed in YPD with increasing concentrations of NAM ± 2 mM Auxin. Growth was normalized to the untreated control.

### Analysis of origin firing by BrdU immunoprecipitation

Cultures were synchronized in G1 with α-factor at 30°C. 30 minutes prior to release, 400 ug/mL BrdU was added to cultures except for the control condition. Release was carried out by addition of 50 μg/mL pronase and 200 mM hydroxyurea and cells were incubated for 90 minutes at 30°C. 0.1% sodium azide was added and cultures were incubated for 10 minutes on ice. Cells were centrifugated and pellets were washed once with TBS, transferred to screwcap tubes and frozen on dry ice. Immunoprecipitation (IP) was performed as previously described [112]. Quantitative PCR was done using 2x SYBR Green qPCR Master Mix (Bimake) per the manufacturer’s guidelines. qPCR plates were analyzed on a ABI7500 real-time PCR system. Since replication at origins doubles the amount of DNA, normalizing on the input signal from probed origins might reduce BrdU signals from replicated regions, and interfere with quantification of changes in origin activity. To remove the contribution of replicated DNA from qPCRs quantifications, we instead normalized IP signals to a region that is expected to remain unreplicated throughout our experiments. We chose the *ACT1* locus that is located 15 kb and 54kb away from the closest origin of replication (ARS603) and telomere, respectively. Replication forks are expected to travel ~3–7.5kb away from origins in our experiments [113], and are therefore not expected to reach the *ACT1* locus. Data from replicate experiments were further normalized to a highly efficient replication origin, ARS305, to account for differences in BrdU incorporation between strains. Data are represented as values relative to the WT strain. Primer pairs are listed in S1 Table.

### *CAN1* mutagenesis assay

6 colonies of relevant genotype were grown to saturation for 48 hours in YPD containing 0.001% MMS. Cells were plated on YPD assess the number of colony forming units and on synthetic media containing 60 μg/mL canavanine to determine the number of *CAN1* mutation events. The frequency of canavanine resistance was calculated as the ratio between colonies growing on canavanine plates and the initial number of plated viable cells (assessed by plating appropriate dilutions on YPD plates). The median *CAN1* mutation frequency of the 6 clones was then determined and data is represented as the average of the median from several experiments. Statistical significance was determined using two-tailed student’s T-tests.

## Supporting information

S1 Fig Supplementary genetic analyses of the sensitivity of *yku70Δ/80Δ* mutants to NAM. (A) *yku70Δ* does not display synthetic growth defects with single sirtuin mutants. (B-C) Preventing H3K56 acetylation rescues the growth of *yku70Δ* and *yku80Δ* mutants in NAM. (D) *fob1Δ* does not rescue the growth defects of *yku70Δ* in NAM (E) Inhibition of the Sir2-Sir3-Sir4 complex is not responsible for the sensitivity of *yku70Δ* mutants to NAM.
(TIF)

S2 Fig Sensitivity of yku70 mutants to NAM-induced replicative stress involves the inhibition of multiple sirtuins. (A) *hst3Δ hst4Δ yku70Δ* mutants are sensitive to NAM. Growth assay in 96-well plates (see materials and methods). Error bars: standard deviation (B) Deletion of both *SIR2* and *HST1* and/or *SUM1* exacerbates the growth defects of *yku70Δ* cells exposed to MMS-induced replicative stress. Five-fold serial dilutions of cells were spotted on the indicated solid media and incubated at 30°C. (C) Growth defects of *yku70Δ* mutants in NAM are maintained despite the absence of Sir2 and/or Hst1. Growth assay in 96-well plates (see materials and methods). Error bars: standard deviation. (D) *sum1Δ* exacerbates the growth defects of *yku70Δ* cells in MMS in a H4K16ac-dependent manner.
(TIF)

S3 Fig Growth defects of *yku70Δ/80Δ* mutants in NAM do not result from loss of TLC1 binding or extensive ssDNA formation at telomeres. (A) *yku80-135i* cells have longer telomeres than *yku80Δ* cells as determined by southern blotting using a probe recognizing telomeric repeats (see materials and methods for details). (B) The yku80-135i mutation does not lead to growth defects in the presence of NAM. (C) Preventing ssDNA formation at telomeres by deleting *EXO1* does not rescue the growth of *yku70Δ* mutants in NAM. (B-C) Growth assay in 96-well plates (see materials and methods). Error bars: standard deviation.
(TIF)

S4 Fig Yku70 is re-expressed within an hour after auxin removal. Yeast cells from the 96 h time point from Fig 2C and 2D were resuspended in YPD medium without auxin. Yku70-AID-Flag re-expression was monitored by immunoblotting. *: non-specific band from anti-Flag antibody.
(TIF)

S5 Fig Telomere length analysis of cells expressing the CDC13-EST1 fusion. DNA was extracted from samples taken for the experiment shown in Fig 3F and analysed by southern blotting using a probe recognizing telomeric repeats (see materials and methods for details).
(TIF)

S6 Fig NAM inhibits the growth of cells with short telomeres without significantly altering telomere length. (A) *elg1Δ* rescues the growth of *yku70Δ* mutants in NAM. Growth assay in 96-well plates (see materials and methods). Error bars: standard deviation. (B) NAM does not affect telomere length. Asynchronous cells were exposed to 20 mM NAM for 8 hrs at 30°C. Samples were taken prior and after NAM exposure for telomere length analysis by southern blotting using a probe recognizing telomeric repeats (see materials and methods for details).
(TIF)

S7 Fig Elevated number of extrachromosomal origins of replication sensitizes cells replicative stress. (A-B) Wild-type cells harbouring YEPFAT 7.5 *leu2-d* plasmids present growth defects upon NAM (A) or MMS (B) -induced replicative stress. (A) Growth assay in 96-well plates (see materials and methods). Error bars: standard deviation. (B) Five-fold serial dilution of cells were spotted on the indicated solid media and incubated at 30°C.
(TIF)

S8 Fig Rad53-dependent inhibition of origin firing in response to replicative stress does not contribute to the growth defects of *yku70Δ* cells exposed to NAM. Growth assay in 96-well plates (see materials and methods). Error bars: standard deviation.
(TIF)

S1 Table Primers used for qPCR. (PDF)
