# Supplementary figures and images for: An interplay between multiple sirtuins promotes completion of DNA replication in cells with short telomeres
Source: PLoS Genet. 2018 Apr 16;14(4):e1007356. doi: 10.1371/journal.pgen.1007356 (PMC5919697; doi:10.1371/journal.pgen.1007356)

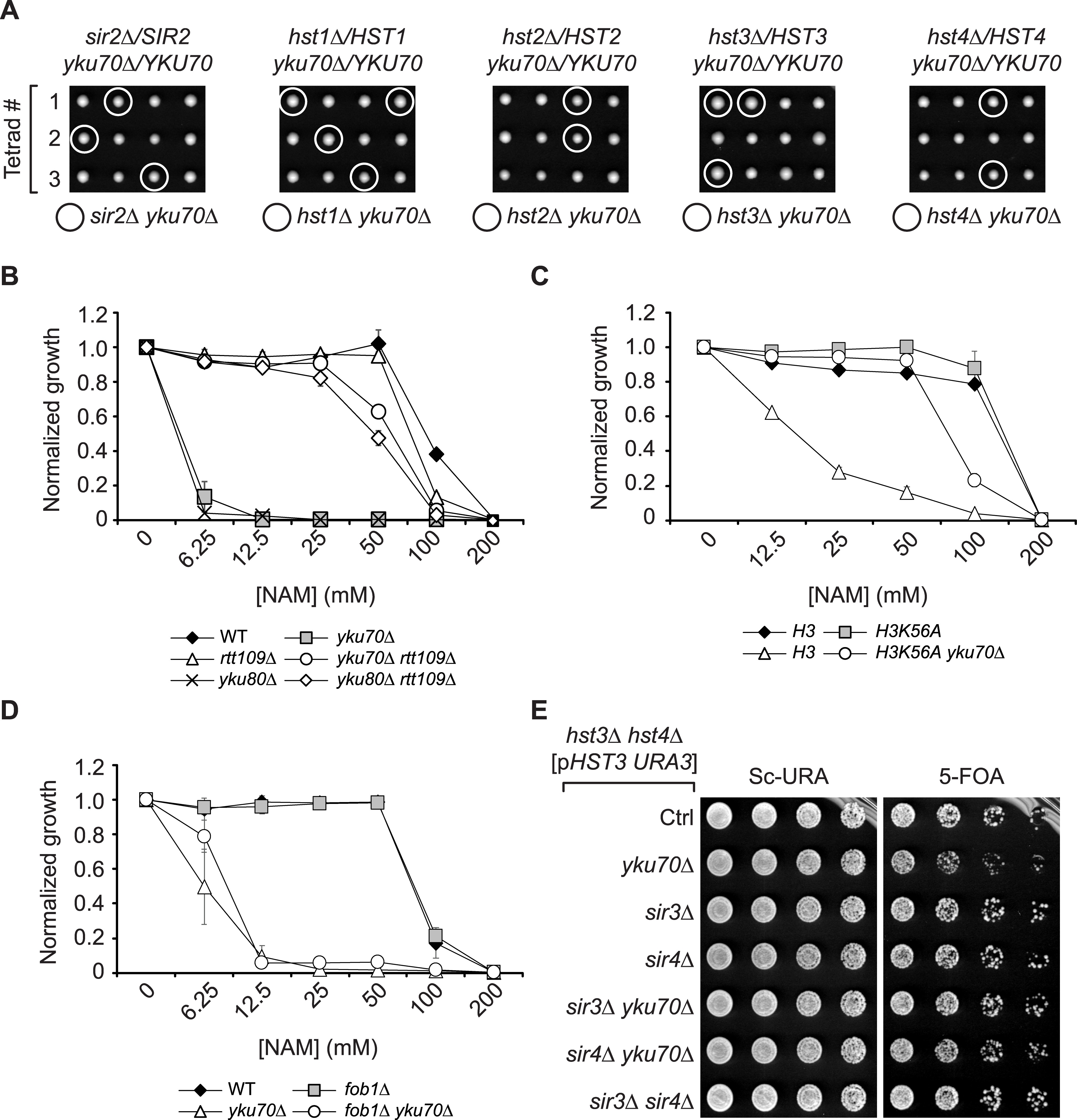

Supplement: S1 Fig — (A) yku70Δ does not display synthetic growth defects with single sirtuin mutants. (B-C) Preventing H3K56 acetylation rescues the growth of yku70Δ and yku80Δ mutants in NAM. (D) fob1Δ does not rescue the growth defects of yku70Δ in NAM (E) Inhibition of the Sir2-Sir3-Sir4 complex is not responsible for the sensitivity of yku70Δ mutants to NAM. (TIF) [file pgen.1007356.s001.tif]

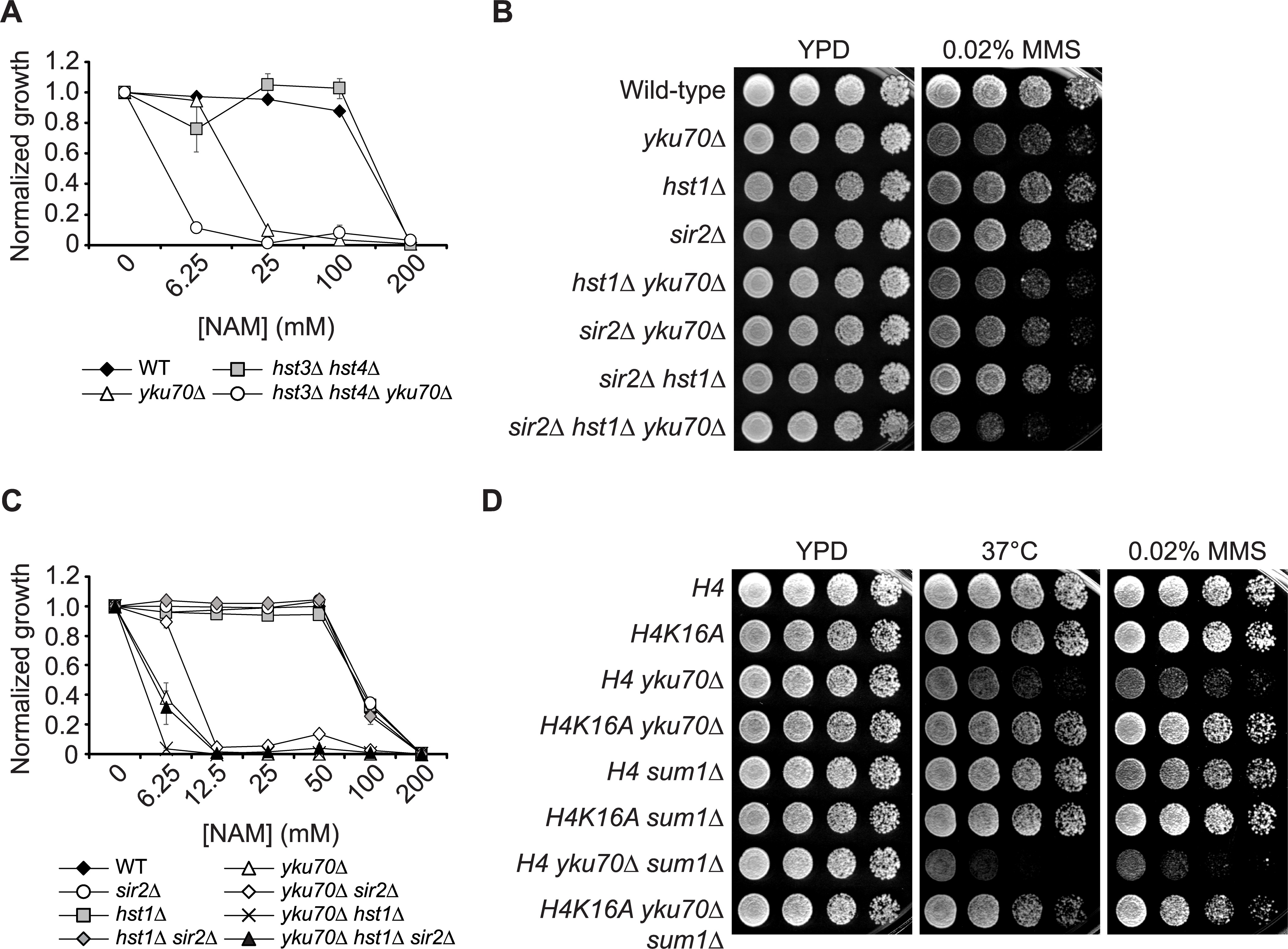

Supplement: S2 Fig — (A) hst3Δ hst4Δ yku70Δ mutants are sensitive to NAM. Growth assay in 96-well plates (see materials and methods). Error bars: standard deviation (B) Deletion of both SIR2 and HST1 and/or SUM1 exacerbates the growth defects of yku70Δ cells exposed to MMS-induced replicative stress. Five-fold serial dilutions of cells were spotted on the indicated solid media and incubated at 30°C. (C) Growth defects of yku70Δ mutants in NAM are maintained despite the absence of Sir2 and/or Hst1. Growth assay in 96-well plates (see materials and methods). Error bars: standard deviation. (D) sum1Δ exacerbates the growth defects of yku70Δ cells in MMS in a H4K16ac-dependent manner. (TIF) [file pgen.1007356.s002.tif]

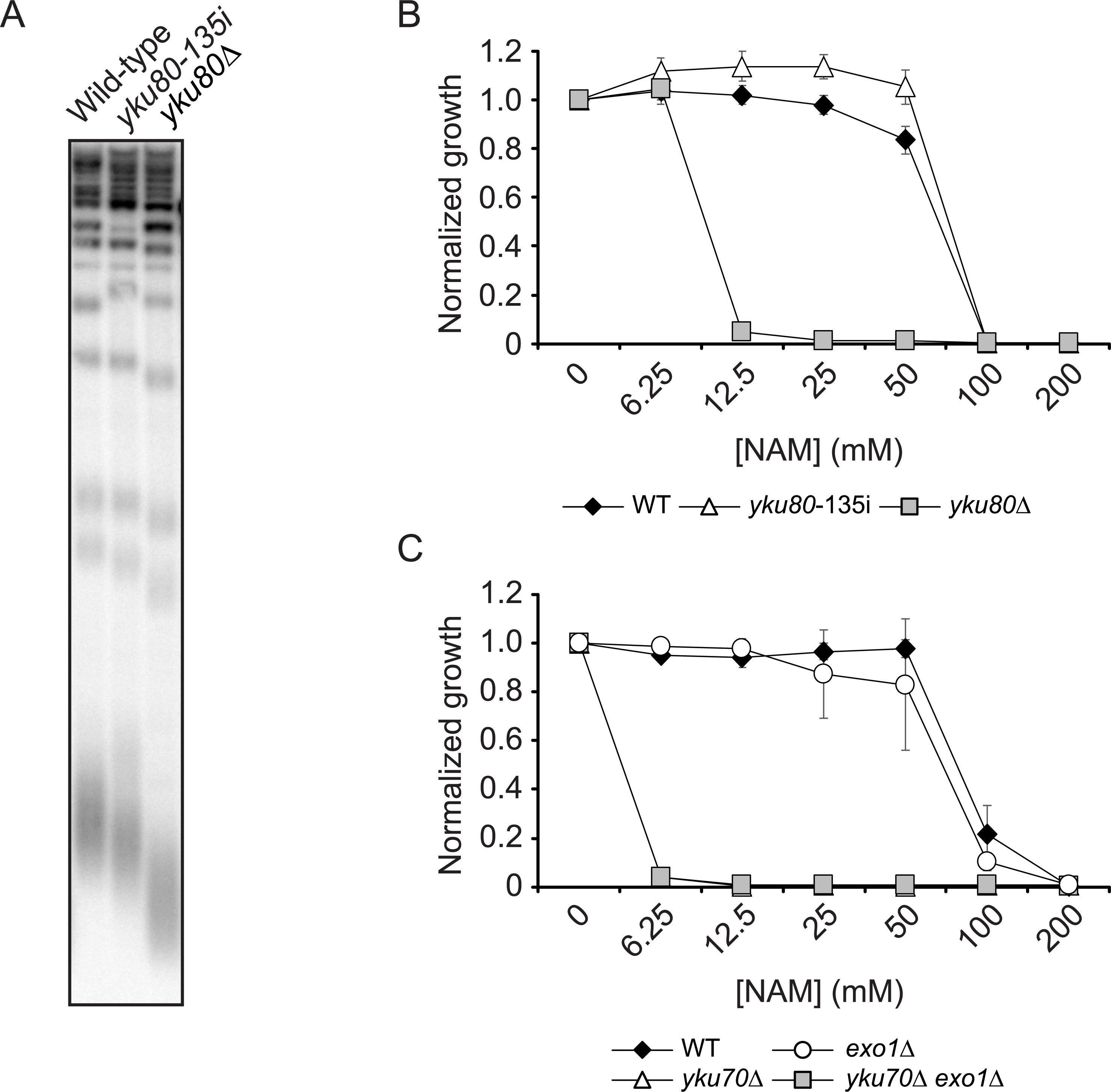

Supplement: S3 Fig — (A) yku80-135i cells have longer telomeres than yku80Δ cells as determined by southern blotting using a probe recognizing telomeric repeats (see materials and methods for details). (B) The yku80-135i mutation does not lead to growth defects in the presence of NAM. (C) Preventing ssDNA formation at telomeres by deleting EXO1 does not rescue the growth of yku70Δ mutants in NAM. (B-C) Growth assay in 96-well plates (see materials and methods). Error bars: standard deviation. (TIF) [file pgen.1007356.s003.tif]

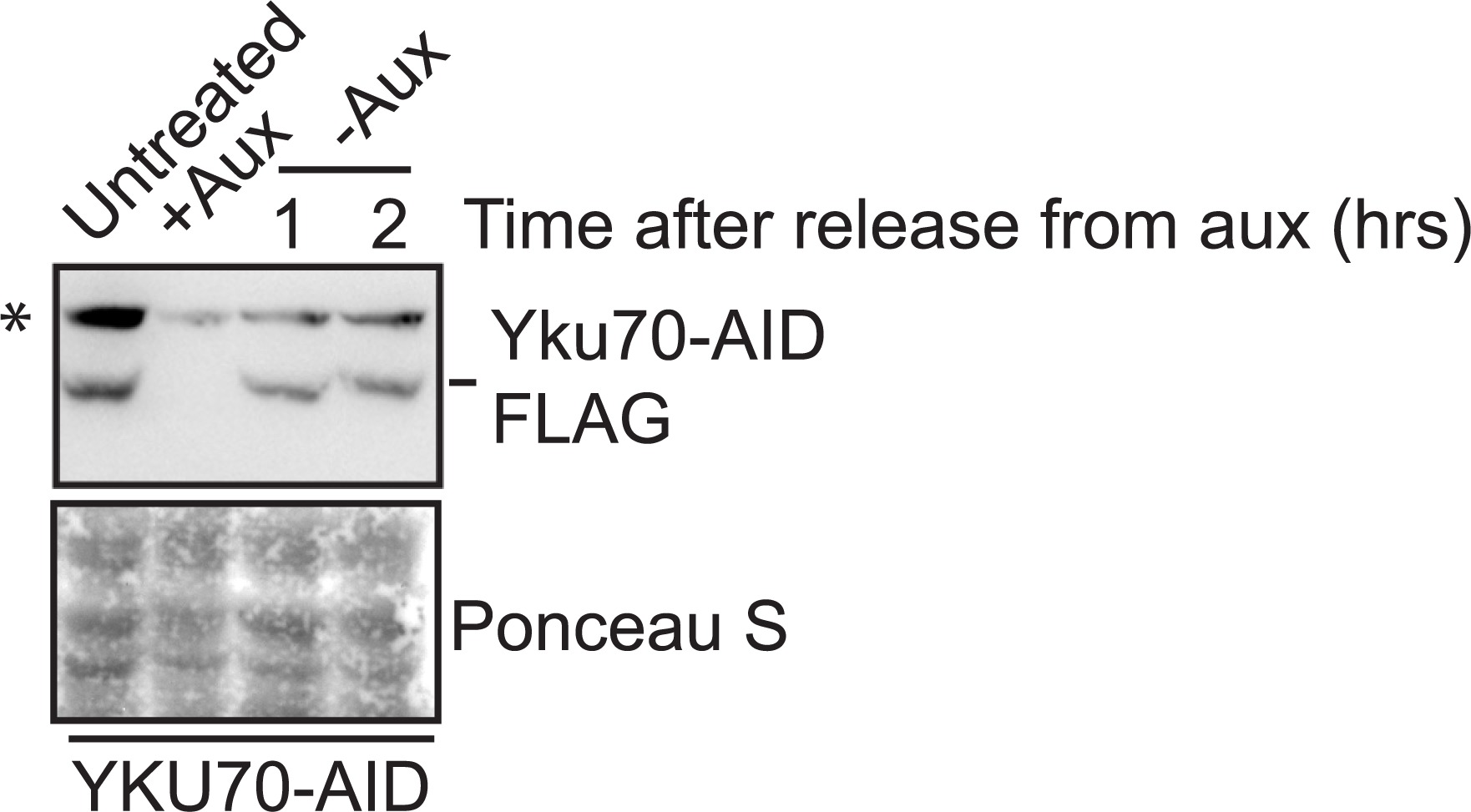

Supplement: S4 Fig — Yeast cells from the 96 h time point from Fig 2C and 2D were resuspended in YPD medium without auxin. Yku70-AID-Flag re-expression was monitored by immunoblotting. *: non-specific band from anti-Flag antibody. (TIF) [file pgen.1007356.s004.tif]

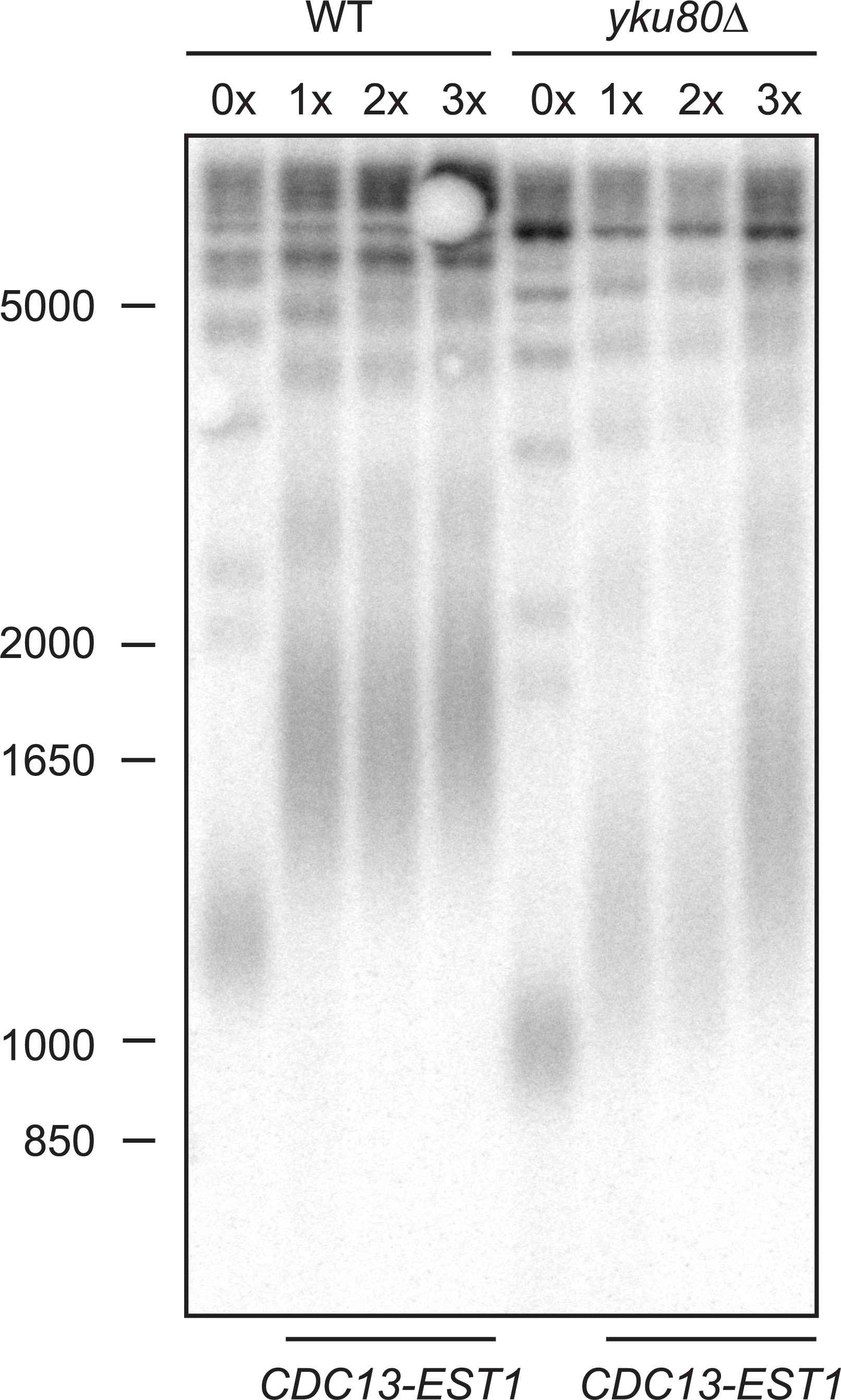

Supplement: S5 Fig — DNA was extracted from samples taken for the experiment shown in Fig 3F and analysed by southern blotting using a probe recognizing telomeric repeats (see materials and methods for details). (TIF) [file pgen.1007356.s005.tif]

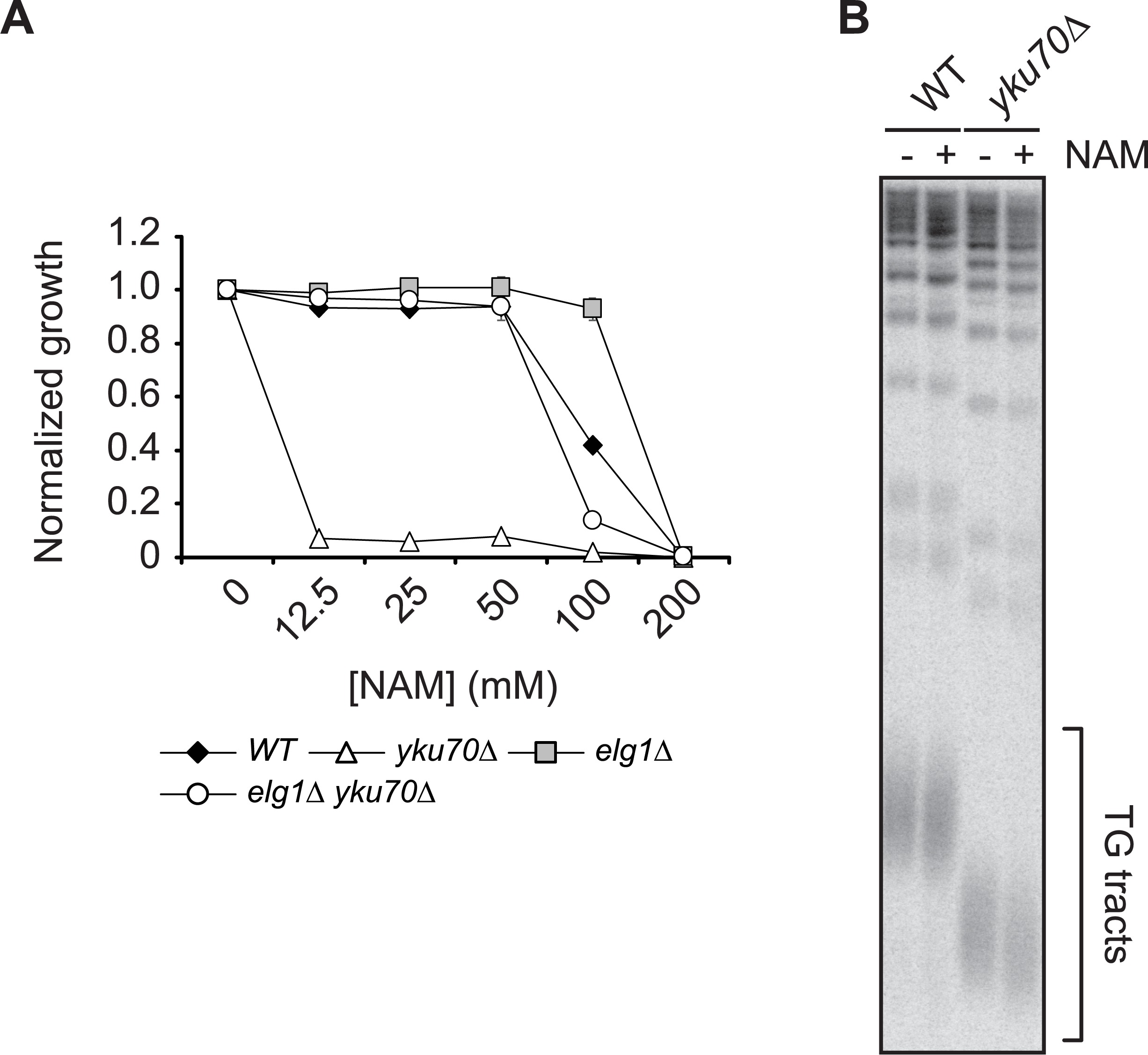

Supplement: S6 Fig — (A) elg1Δ rescues the growth of yku70Δ mutants in NAM. Growth assay in 96-well plates (see materials and methods). Error bars: standard deviation. (B) NAM does not affect telomere length. Asynchronous cells were exposed to 20 mM NAM for 8 hrs at 30°C. Samples were taken prior and after NAM exposure for telomere length analysis by southern blotting using a probe recognizing telomeric repeats (see materials and methods for details). (TIF) [file pgen.1007356.s006.tif]

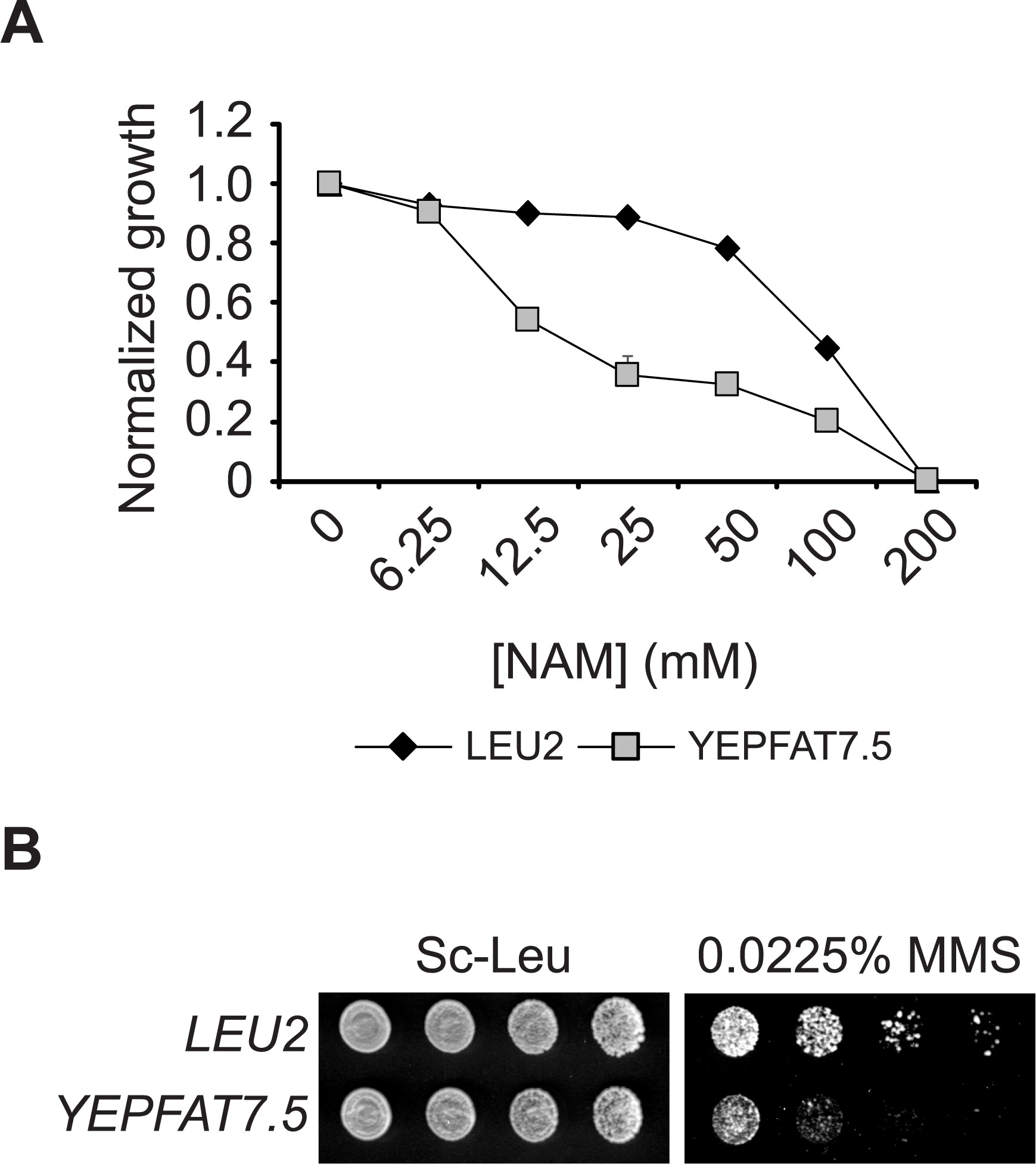

Supplement: S7 Fig — (A-B) Wild-type cells harbouring YEPFAT 7.5 leu2-d plasmids present growth defects upon NAM (A) or MMS (B) -induced replicative stress. (A) Growth assay in 96-well plates (see materials and methods). Error bars: standard deviation. (B) Five-fold serial dilution of cells were spotted on the indicated solid media and incubated at 30°C. (TIF) [file pgen.1007356.s007.tif]

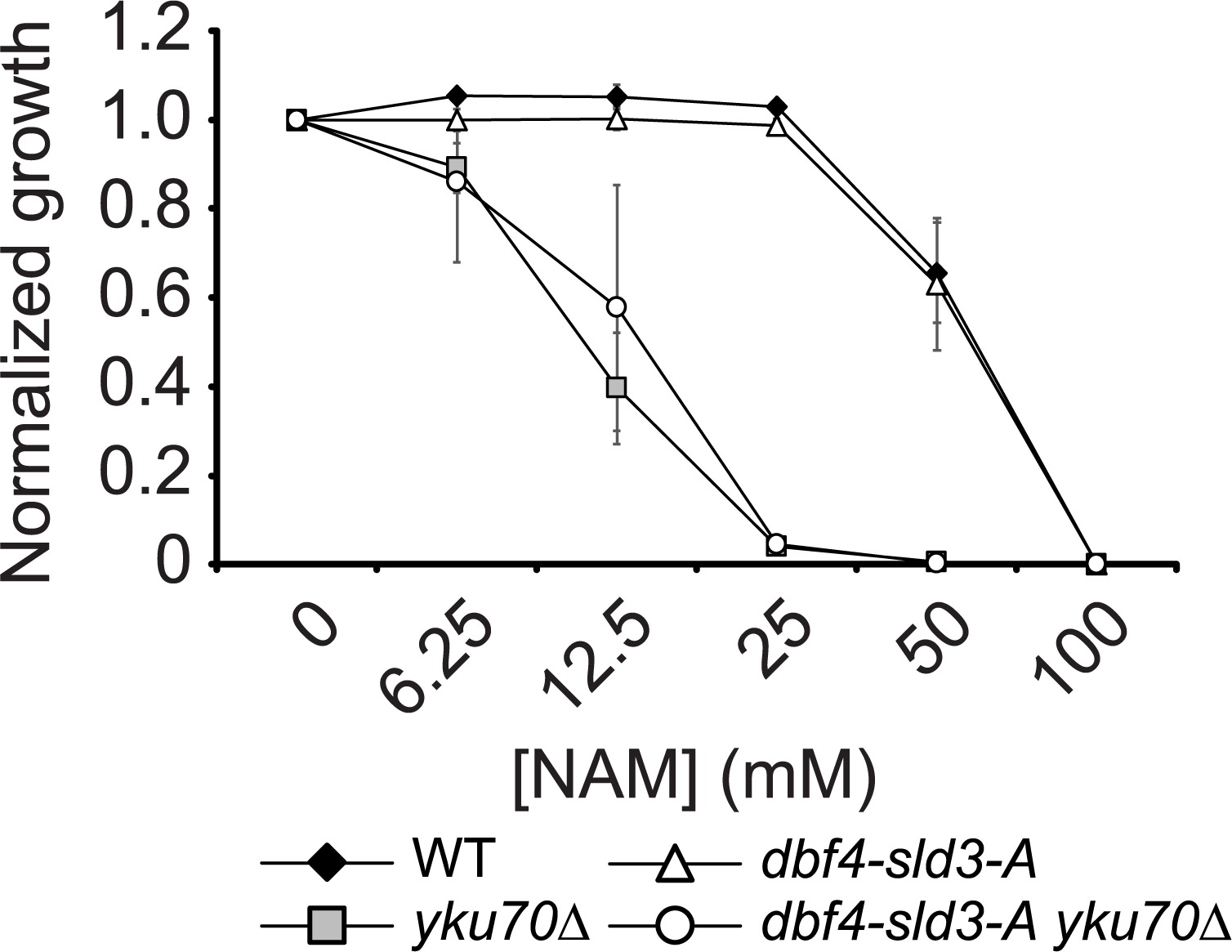

Supplement: S8 Fig — Growth assay in 96-well plates (see materials and methods). Error bars: standard deviation. (TIF) [file pgen.1007356.s008.tif]
