# Supplementary material for: An interplay between multiple sirtuins promotes completion of DNA replication in cells with short telomeres
Source: PLoS Genet. 2018 Apr 16;14(4):e1007356. doi: 10.1371/journal.pgen.1007356 (PMC5919697; doi:10.1371/journal.pgen.1007356)
Supplement: S1 Table — (PDF) [file pgen.1007356.s009.pdf]

S1 Table. Primers used for qPCR

Name	Sequence
TEL01L-F	CGGTGGGTGAGTGGTAGTAAGTAGA
TEL01L-R	CATCCTAACACTACCCTAACACAG
ARS522-F	GGCAGATGGTAGGAAACCAA
ARS522-R	TCAGGCATATCTGGGGAATC
ARS305-F	CGCCTTTTGACAGGACGATA
ARS305-R	GCTCCAAAGAAGGCTCTGAA
ACT1-F	TCGTTCCAATTTACGCTGGTT
ACT1-R	CGGCCAAATCGATTCTCAA
ARS610-F	AGCGGCTGGACTACTTTCTGGAAT
ARS610-R	GCATCCACTCGTTAGGATCACGTT
